# Trinuclear Magnesium Imidazolate Borohydride Complex

**DOI:** 10.1021/acs.inorgchem.2c01319

**Published:** 2022-08-02

**Authors:** Maja Reberc, Matjaž Mazaj, Jernej Stare, Marta Počkaj, Gregor Mali, Xiao Li, Yaroslav Filinchuk, Radovan Černý, Anton Meden

**Affiliations:** † Faculty of Chemistry and Chemical Technology, University of Ljubljana, Večna pot 113, 1001 Ljubljana, Slovenia; ‡ National Institute of Chemistry, Hajdrihova 19, 1000 Ljubljana, Slovenia; ¶ IMCN Université Catholique de Louvain, Place L. Pasteur 1, B-1348, Louvain-la-Neuve, Belgium; § DQMP, University of Geneva, 24 quai Ernest-Ansermet, 1211 Geneva, Switzerland

## Abstract

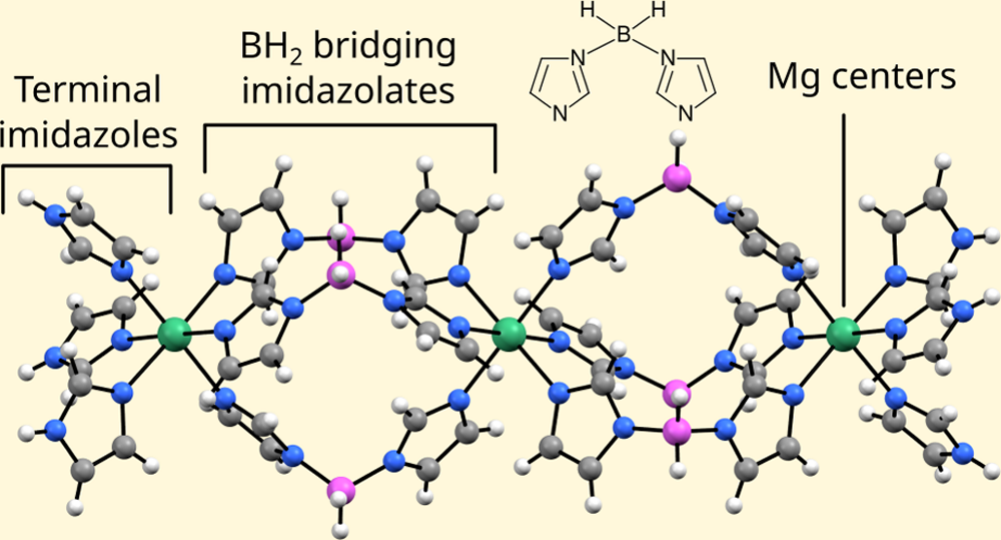

A new type of hybrid compound, combining properties of MOFs and borohydrides, was synthesized solvothermally using Mg(BH_4_)_2_ and imidazole as precursors. Material in the form of acetonitrile solvate with formula [Mg_3_{(Im)BH_2_(Im)}_6_(ImH)_6_]·CH_3_CN crystallizes in the space group *R*3̅, having the unit cell parameters *a* = 15.1942(2) Å and *c* = 28.3157(3) Å as determined by single crystal X-ray diffraction. The structure was further investigated by solid-state NMR and DFT quantum chemical calculations. The main feature of the structure, reported here for the first time, is a linear trinuclear complex, where octahedrally nitrogen-coordinated Mg^2+^ ions are bridged with {(Im)BH_2_(Im)}^−^ units, forming inside voids of 4.6 Å in diameter between the magnesium ions. Polar intermolecular interactions hold the molecules in a dense rhombohedral stacking, where a disordered acetonitrile molecule plays a cohesive role. The compound is stable in air and upon heating to about 160 °C. Using an alternative synthesis method from an imidazole melt, an imidazole solvate with the formula [Mg_3_{(Im)BH_2_(Im)}_6_(ImH)_6_]·ImH and a very similar crystal structure to acetonitrile solvate was prepared. It is stable up to 220 °C. Upon further heating, it transformed into a layered structure with the formula Mg(Im_3_BH)_2_, space group *P*3̅1*c*, and unit cell parameters *a* = 8.7338(9) Å and *c* = 17.621(2) Å determined by synchrotron powder diffraction. Besides its structural novelty, two types of potentially reactive hydrogens, bonded to boron and nitrogen in the same molecule, make the material highly interesting for future investigations in the fields of energy storage applications.

## Abstract 2

A trinuclear complex with magnesium centers, imidazolate−borohydride−imidazolate bridging ligands, and imidazole terminal ligands was synthesized in the form of an acetonitrile solvate and imidazole solvate. The compounds represent a rare case of imidazolyl borates where two potentially reactive hydrides remain on the boron. Heating of the title material leads to the release of hydrogen due to the comproportionation of borohydride and imidazole hydrogen atoms, resulting in a new layered structure of Mg(BHIm_3_)_2_.

## Introduction

Research on hybrid organic–inorganic systems has experienced immense interest in recent decades. The integration of organic and inorganic moieties either on atomic or macroscopic scale can namely synergistically improve the physicochemical properties or even generate features that individual components do not possess. Therefore, this concept has resulted in the development of numerous advanced materials which are considered to be the state-of-the-art in the fields of catalysis,^1−5^ bioapplications,^3,6−9^ electrochemistry,^10,11^ ionic conductivity,^12−15^ sensoring,^16,17^ and energy performance.^18−20^ Among energy storage processes, hydrogen is considered as one of the most promising energy carriers as an alternative to fossil fuels. The biggest challenge, however, represents its storage under low-cost and safe conditions. In that manner, the development of solid materials that would capture hydrogen via physisorption or chemisorption providing sufficiently high gravimetric and volumetric density of hydrogen and suitable adsorption/desorption thermodynamics and kinetics still remains a great obstacle. Most frequently studied materials for adsorption of hydrogen are metal–organic frameworks (MOFs) and metal-based hydrides/borohydrides.^21^ MOFs can, on one hand, possess low-density structures with a highly accessible pore system and tunable structure functionality; however, the majority of the developed MOF systems still suffer from low mass and volume capacity of hydrogen at ambient conditions due to weak framework-to-hydrogen interactions. On the other hand, borohydrides can contain sufficient amounts of hydrogen even at room temperature and pressure, but its covalent bonding inhibits the efficiency of desorption and makes this process irreversible. A possible solution that could overcome the drawbacks of both types of materials is the development of hybrid architectures containing the structural features of both metal borohydrides and metal–organic frameworks. Nanoparticles of various metal borohydrides have already been confined within MOF structures, resulting in improved energy efficiency of intrinsic borohydride dehydrogenation^22−24^ which can either enhance hydrogen release at lower temperatures^25^ or generate catalytically active reducing sites within MOF frameworks.^24,26,27^ However, nanoconfined metal borohydride/MOF composites contain in most cases “extra-framework” borohydride species applied within MOF matrixes mostly under postsynthesis conditions, establishing relatively weak van der Waals-type interactions with matrix frameworks. This can importantly affect the durability of the sorption or catalytic system.

Many structures containing boron atoms, bound to the one of the nitrogen atoms of the imidazolate units in their frameworks, have already been reported. Most of them belong to a subset of MOFs, named BIFs (boron imidazolate frameworks).^28^ These structures feature three or four imidazolate anions or their derivates bound to boron centers via one of the nitrogen atoms, having various mono- and divalent metal cations coordinated to the other nitrogen atom of the imidazolate. Only in the case of trisubstituted boron imidazolates of this kind, containing the unit BH(Im)_3_^–^, is one of the potentially reactive hydrides, which is bound to boron, preserved.

Searching for metal imidazolates hydridoborates with more than one hydride bound to boron in the Cambridge Structural Database (CSD)^29^ revealed very little results. Two hydrides bound to boron are present only in two structures: catena-((μ_2_-dihydrobis(1-imidazolyl)borate)-(tricyclohexylphosphine)-silver(I)), (C_24_H_41_AgBN_4_P)_*n*_, CSD refcode QESTIR, and bis(μ_2_-dihydrobis(1-imidazolyl)borate)-tetrakis(tri-*p*-tolylphosphine)-disilver(I) chloroform solvate, C_96_H_100_Ag_2_B_2_N_8_P_4_·2CHCl_3_, CSD refcode QESTUD.^30^ In both of them the metal atom is silver and imidazolate is unsubstituted (hydrogen atom is bound to each of the three carbon atoms). QESTIR is a chain coordination polymer, while QESTUD contains cyclic molecular units. No structure of this type with three hydrides bound to boron was found, and only three structures with coexisting tetrahydridoborate and a metal, coordinated to the N atom of an imidazole, could be found in the CSD. These are molecular (2,2,13,13-tetraisopropyl-3a,11a,14a,16b-tetrahydro-4*H*,11*H*-[1,3]dioxolo[3,4]pyrrolo[1,2-*a*][1,3]dioxolo[3″,4″]pyrrolo[2″,1″:2′,3′]imidazo[4′,5′:7,8]naphtho[1,2-*d*]imidazole)-bis(tetrahydroborate)-cobalt(II) diethyl ether solvate, C_32_H_48_B_2_CoN_4_O_4_·C_4_H_10_O, CSD refcode NUFXOD,^31^ chain catena-[tris(*m*-1,1′-[1,4-phenylenebis(methylene)]di(1*H*-imidazole))-tetrakis(tetrahydroborate)-dimanganese(II)], (C_42_H_58_B_4_Mn_2_N_12_)_*n*_, CSD refcode SOHHEF,^22^ and molecular tris(*N*-methylimidazole)-tetrahydroborato-lithium, C_12_H_22_BLiN_6_, CSD refcode SUMZEG.^32^

The first compound with simultaneous presence of deprotonated imidazolate, acting as N-donor ligand, and tetrahydridoborate was prepared in 2017 by Morelle, who synthesized the compound Li_2_ImBH_4_. It has three known polymorphic forms, all showing extended (layer or framework) structural features.^33^ Besides that, similar derivatives were obtained from LiBH_4_ and methyl- and benzimidazolates of Li. To our knowledge, these three compounds are the only known examples of this kind up to now. The compound with benzimidazolate, Li_2_(bIm)BH_4_, shows remarkable dynamics of groups (rotational tunneling), favored by its unusual environment.^34^

During efforts to produce similar compounds with other light metals, we were able to synthesize a previously unknown molecular compound, which is reported in this work. Because the compound is new, most efforts were devoted to thorough structural characterization, using single crystal X-ray diffraction, solid-state NMR, and quantum chemical calculations, as we wanted to clearly define the identity of the new material and provide the basis for understanding its properties. In addition, we aimed to determine some basic physicochemical properties of the novel compound such as stability in air, thermal stability, and sorption properties.

In search of an alternative method of synthesis with the intention of producing a solvent-free form of the title compound, a procedure using molten imidazole was found. It, interestingly, resulted in an imidazole solvate, which we also report here.

## Experimental Section

### Material Synthesis

The compound in the form of acetonitrile solvate was prepared using the solvothermal synthesis method inside a glovebox with nitrogen atmosphere. Magnesium borohydride (Mg(BH_4_)_2_, Sigma-Aldrich, 95%), imidazole (ImH, Sigma-Aldrich, ≥99%), and acetonitrile (CH_3_CN, Sigma-Aldrich, 99.8%, dried over CaH_2_) were added to a 23 mL Teflon-lined stainless steel autoclave, which was then heated in an oven at 85 °C for 24 h. The product was centrifuged at 5000 rpm for 5 min in the glovebox. The supernatant was decanted, and fine white powder was obtained. After the remaining solvent evaporated from the Teflon liner, colorless crystals formed on its surface. A more detailed description of the procedure and investigated reactant ratios are available in the Supporting Information.

Another procedure that avoids the use of autoclaves and solvents, aiming to produce a solvent-free compound, was subsequently developed, namely, synthesis in imidazole melt. It was found that the product is not solvent-free. However, it is different from the acetonitrile solvate, and it is therefore worth reporting its synthesis. γ-Mg(BH_4_)_2_ (272 mg, 5 mmol) and imidazole (2720 mg, 40 mmol) were loaded into a modified Schlenk flask in a glovebox with argon atmosphere. The flask was connected to the Schlenk line, and the mixture was heated in a flow of argon in an oil bath until the solid imidazole was molten at about 70–85 °C. Reaction was manifested by the evolution of hydrogen in the form of bubbles. The temperature of the reaction mixture was kept at 110 °C for 20 h. The heating was stopped, and the product was washed with tetrahydrofuran (THF) while hot, aiming to remove the excess imidazole. Twenty milliliters of THF was used at first, and then the product was rinsed three more times with 8 mL of THF. The product was dried under vacuum at room temperature.

### Structural Characterization

#### Single-Crystal X-ray Diffraction

A single crystal of the title compound in the form of an acetonitrile solvate was dipped into silicon grease, mounted onto the tip of a glass fiber, and transferred to the goniometer head, under a nitrogen cryostream. Data were collected on a SuperNova diffractometer equipped with an Atlas detector, using CrysAlis PRO software, and monochromated Cu Kα radiation (1.54184 Å) at 150 K.^35^ The initial structural model containing the coordination complex was obtained using the Superflip structure solution program.^36^ Full-matrix least-squares refinement on *F*^2^ with anisotropic displacement parameters for all nonhydrogen atoms was carried out using SHELXL-2018/3.^37^ All H atoms were initially located in difference Fourier maps and were further treated as riding on their parent atoms with C(aromatic)–H = 0.95 Å. Hydrogens, bonded to B, were refined freely, while the N–H bonds were restrained to 0.87(2) Å.

The atoms forming the acetonitrile molecules were observed in the difference Fourier maps. The obtained molar ratio between [Mg_3_{(Im)BH_2_(Im)}_6_(ImH)_6_] and acetonitrile molecules was 1:1, resulting in molecular formula C_54_H_72_B_6_Mg_3_N_36_·C_2_H_3_N. The central atom of the acetonitrile, C11, resided on an inversion center positioned on a 3-fold inversion axis, which led to severe symmetry-induced disorder of the acetonitrile molecules with ill-defined bond lengths and angles, and precluded the location of hydrogen atoms. Consequently, the coordinates of acetonitrile molecule were taken from the results of quantum chemical calculations, from the model with the lowest potential energy, and they were fixed in the last refinement cycles while atomic displacement parameters of all acetonitrile atoms were refined isotropically. In the trials to refine the ADPs of the acetonitrile atoms anisotropically, some of them became nonpositive definite. For the acetonitrile hydrogens, their isotropic displacement parameters were restrained to be 1.5-times larger than that of their carrier atoms.

Figures depicting the structure were prepared with Mercury.^38^ Details on crystal data, data collection, and structure refinement, as well as data on selected bond lengths and angles, are given in the Supporting Information.

#### Crystal Structure Optimizations

Periodic DFT calculations were employed for the assessment of various aspects of the structure of the title system in the form of acetonitrile solvate. Optimization of the structures was carried out with the VASP 5.3.5 program package^39−42^ using the revised version^43^ of the Perdew–Burke–Ernzerhof functional,^44^ corrected for dispersion interactions by the zero damping DFT-D3 method of Grimme,^45^ plane-wave basis set with a kinetic energy cutoff of 500 eV, Projector Augmented Wave atomic pseudopotentials,^46,47^ and Monkhorst–Pack^48^ k-point mesh of 2 × 2 × 1 points. Structures subject to optimization were built on the basis of the present crystal structure solution by using the CIF2Cell utility.^49^ Various optimization strategies were imposed, differing mainly in fixation/relaxation of unit cell parameters and adjusting optimization step width scaling constant. When using fixed unit cell parameters, the corresponding values were set to those obtained by diffraction.

The crystal structure of the title system was found to contain disordered molecules of acetonitrile solvent, with the central carbon atom of the linear C–C–N moiety positioned nearly on a 3-fold inversion axis. According to crystallography, this would generate six different orientations of the molecule. The CH_3_CN molecule is linear, and from the results of X-ray diffraction it could be concluded that it is not perpendicular to the 3-fold axis but encloses an angle of about 80° with it. The 3-fold inversion axis first generates two additional molecules in increments of 120°. All three crystallographically equivalent molecules are then inverted by the same symmetry element, bringing the terminal C atom nearly over the N atom and vice versa. From the view along the 3-fold inversion axis, it appears to have six molecules in increments of 60°, forming a hexagon, while the side view reveals that three of them have an inclination to the 3-fold axis of about 80°, and the other three are inclined by about 100°. All six orientations were investigated with calculations. Accordingly, crystal structures corresponding to these orientations (oriented identically in all three occurrences in the unit cell) were prepared in space group *P*1 and optimized using the settings given above. Additionally, all solvent molecules were removed from the crystal structure, which was then optimized, focusing on the change in intermolecular interactions and in the density. Finally, a single acetonitrile molecule in vacuum and the structure model with acetonitrile molecules removed were optimized separately to estimate the interaction energy of acetonitrile with the rest of the structure. The acetonitrile molecule was placed in a cubic box of 20 Å. Periodicity was still imposed, but the large size of the box ensures that intermolecular interactions become vanishingly small, thereby reasonably mimicking a vacuum model.

Prior to above calculations, a structure with a single orientation of acetonitrile molecules and an identical model structure with acetonitrile molecules removed were optimized using three different functionals: the original Perdew–Burke–Ernzerhof^44^ (PE), its revised version^43^ (RP), and a version specially tuned for solids^50^ (PS). Of those, functional RP was identified as optimal on the criterion of match between optimized and experimental unit cell parameters (Table S4 in the Supporting Information) and was used for all following calculations.

#### Rietveld Analysis of Powder Patterns

Rietveld analysis was performed using TOPAS-Academic V7.^51^ The structural model of the [Mg_3_{(Im)BH_2_(Im)}_6_(ImH)_6_] moiety was constructed in the form of a *Z*-matrix based on the crystal structure determined by single-crystal X-ray diffraction. The acetonitrile and imidazole molecules and the [BIm_3_H] moiety were also constructed in the form of *Z*-matrixes using the interatomic distances and angles found for similar fragments in the CSD. The orientations and positions of the solvent molecules were freely refined, as was the rotation of the [Mg_3_{(Im)BH_2_(Im)}_6_(ImH)_6_] moiety about the 3-fold axis, while the position and orientation of the latter along the axis were constrained by keeping the central magnesium atom on the inversion center at the 3-fold axis and the other two Mg atoms (which are symmetry equivalents) on the 3-fold axis but off the inversion center. All distances, angles, and dihedral angles were then refined with restrictions that kept the molecular geometries within acceptable limits, derived from the structures of similar fragments found in the CSD. For more details, see the Supporting Information.

#### Liquid-State NMR

The nuclear magnetic resonance (NMR) spectrum of the imidazole solvate was measured on a Bruker 500 UltraShield spectrometer. The compound was dissolved in deuterated water (D_2_O) and immediately characterized by ^11^B NMR spectroscopy.

#### Solid-State NMR

Solid-state NMR spectra of the title system in the acetonitrile solvate form were measured on a 600 MHz Varian VNMRS spectrometer, equipped with a 1.6 mm HXY CPMAS probe. Larmor frequencies for ^1^H, ^11^B, and ^13^C nuclei were 599.51, 192.34, and 150.76 MHz, respectively. All measurements, except ^1^H MAS NMR measurement, were carried out at a sample rotation frequency of 20 kHz. The ^1^H MAS NMR spectrum was obtained at a sample rotation frequency of 40 kHz, using a 90° excitation pulse of 1.4 μs and repetition delay of 10 s. ^11^B MAS NMR measurement employed a short 0.8 μs excitation pulse and high-power XiX proton decoupling during acquisition; 128 scans were collected, and the delay between the scans was 10 s. ^1^H–^13^C LG-CPMAS experiment used Lee–Goldburg cross-polarization scheme with duration of 100 μs and high-power proton decoupling; the repetition delay was 2 s, and the number of scans was 4800. In both 2D HETCOR experiments spectral width and number of increments in the indirect dimension were 20 kHz and 20, respectively, XiX decoupling was used, and repetition delay was 2 s. For the measurement of the ^1^H–^11^B HETCOR spectrum, a constant-amplitude cross-polarization block of 80 μs was used and the number of scans was 40; for the ^1^H–^13^C HETCOR spectrum, a ramped-amplitude cross-polarization block of 2 ms was employed (ramp on the proton channel) and the number of scans was 800. Chemical shifts of ^1^H and ^13^C nuclei were reported relative to the corresponding signals of tetramethylsilane, whereas the ^11^B chemical shift axis was set using a 1 M solution of H_3_BO_3_ in water as a secondary reference (^11^B nuclei resonate at 19.6 ppm).

#### NMR Calculations

First-principles calculations of chemical-shielding and quadrupolar-coupling parameters were carried out with the GIPAW/DFT approach using CASTEP software package (Materials Studio v. 5.5.3, Accelrys Software Inc.). Two different structural models were derived from the XRD-based model, one without acetonitrile molecules and with an *R*3̅ space group, and one with acetonitrile molecules and with a *P*1 space group. Both structural models were geometry optimized with the DFT-based structure relaxation. Plane-wave basis, generalized gradient approximation of Perdew–Burke–Ernzerhof and ultrasoft pseudopotentials (generated on-the-fly with CASTEP) were employed. Two runs of geometry optimizations were carried out for each model, one with and one without allowing the variation of the unit cell parameters. Upon optimization, the force on each atom was smaller than 0.08 eV/Å and the stress was below 0.1 GPa. In the DFT-based structure relaxation, the kinetic-energy cutoff for the plane-wave basis was set to 600 eV, and the reciprocal-space sampling was performed with a k-point grid spacing of 0.1 Å^–1^ or less. For the calculations of chemical shifts and quadrupolar coupling constants, the kinetic-energy cutoff for the plane-wave basis was increased to 700 eV and the k-point grid spacing was decreased below 0.05 Å^–1^. GIPAW calculations yielded isotropic chemical shielding σ^iso^, from which the isotropic chemical shift was obtained as δ_CS_^iso^ = σ^ref^ – σ^iso^. Here σ^ref^ was 168 ppm for ^13^C and 30 ppm for ^1^H nuclei. Agreement between the calculated and the experimentally determined chemical shifts was slightly better for structural models, in which only variation of atomic coordinates was allowed.

### Stability, Morphology, Purity, and Sorption Measurements

#### Scanning Electron Microscopy

SEM observations were performed on a Zeiss Supra 3VP field-emission gun scanning electron microscope (FEG-SEM).

#### Powder X-ray Diffraction

Samples of the acetonitrile solvate were finely ground and loaded in glass capillaries (Hilgenberg, diameter 0.7 mm), which were broken off above sample level and sealed with modeling clay. Measurements of transmission geometry were carried out with a PANalytical X’Pert Pro MPD powder diffractometer using Cu Kα_1_ radiation (45 kV, 40 mA) in the range 7–33°2θ with a step time 800 s and a step size 0.05°2θ. The powder phase and predominantly single-crystal phase of most syntheses were analyzed separately. In addition, a powdered sample was analyzed after 29 days of exposure to air to study stability of the title compound. Reflection geometry with Cu Kα_1,2_ radiation (45 kV, 40 mA) was used in the range 3–50°2θ with a step time 100 s and a step size 0.034°2θ.

The temperature-programmed X-ray powder diffraction pattern of powdered samples of the acetonitrile solvate was recorded also on the PANalyticalX’Pert PRO diffractometer additionally equipped with a high-temperature sample cell, from room temperature to 700 °C in steps of 20 °C in static air.

All temperature-programmed measurements of the imidazole solvate of the title compound were performed at SNBL/ESRF. A Pilatus 2 M detector was used with a wavelength 0.728030 Å.

#### Thermal Analysis

Thermal gravimetric analysis (TGA) was performed using a Q5000 IR thermogravimeter (TA Instruments, Inc.). The measurements were carried out in an airflow (10 mL/min) at a heating rate of 10 °C/min.

#### Infrared Spectroscopy

A powdered sample was exposed to air and periodically analyzed by infrared spectroscopy to study stability. The spectra were acquired using Bruker Alpha II and PerkinElmer Spectrum Two FTIR spectrometers in ATR configuration.

#### Sorption Measurements

N_2_ and H_2_ sorption isotherms were measured with an HTP-IMI analyzer (Hiden Isochema Inc.). Before the measurements, the sample was outgassed at 50 °C for 2 h.

## Results and Discussion

### Synthesis

Borohydride-type materials are usually produced without solvent using a mechanochemical process due to the reactivity of borohydride precursors and yield only finely powdered products. This approach therefore makes the structure analysis more difficult. The facile solvothermal approach described herein enables the growth of larger crystals suitable for single-crystal structure analysis.

Product with the formula [Mg_3_{(Im)BH_2_(Im)}_6_(ImH)_6_]·CH_3_CN and rather large crystallites was synthesized from Mg(BH_4_)_2_, ImH, and CH_3_CN in a molar ratio of 1:15:680 (15.2 mg, 287.5 mg, and 10 mL, respectively) in a yield of 55% referring to Mg(BH_4_)_2_. A SEM micrograph of a crystal obtained is shown in Figure S1 in the Supporting Information.

The imidazole solvate [Mg_3_{(Im)BH_2_(Im)}_6_(ImH)_6_]·ImH (molecular formula C_54_H_72_B_6_Mg_3_N_36_·C_3_H_4_N_2_) was obtained in the form of a white powder from Mg(BH_4_)_2_ and ImH melt. The yield was 53% (1.2 g).

In both cases, a reaction between BH_4_^–^ ions and imidazole molecules was observed, releasing hydrogen and forming imidazolyl borates, which were later consumed as ligands in the formation of the trinuclear complex. The reaction occurred at ambient pressure, immediately after the reactants (Mg(BH_4_)_2_ and imidazole) were in a liquid medium. In acetonitrile this occurred at room temperature, and in molten imidazole shortly after it melted.

### Structural Characterization of the Acetonitrile Solvate

**Figure 1:**
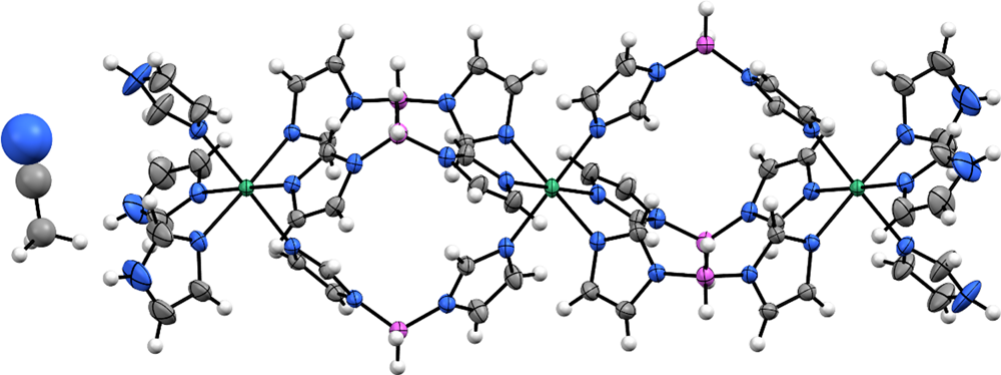
Structure of the [Mg_3_{(Im)BH_2_(Im)}_6_(ImH)_6_] trinuclear complex and disordered acetonitrile (only one of six acetonitrile symmetry-related overlapping positions is shown for clarity). Mg atoms, green; N atoms, blue; C atoms, gray; B atoms, pink; H atoms, white.

The crystal structure of the acetonitrile solvate was successfully solved using single-crystal XRD from a rod-shaped crystal with a typical size of 50 × 200 μm. X-ray structural analysis on a single crystal has shown that the title compound is composed of Mg-based trimeric coordination complexes (Figure 1) parallel to the crystallographic *c* direction and the solvent acetonitrile molecules. The unit cell is given in Table 1.

**Table 1:** Unit Cell Parameters and Some Related Crystallographic Properties of the Reported Structures^a^

structure	SG	*a* [Å]	*c* [Å]	*V* [Å^3^]	*Z*
[Mg_3_{(Im)BH_2_(Im)}_6_(ImH)_6_]·CH_3_CN	*R*3̅	15.1942(2)	28.3157(3)	5661	3
[Mg_3_{(Im)BH_2_(Im)}_6_(ImH)_6_]·ImH	*R*3̅	15.3943(5)	28.4441(18)	5838	3
Mg(BHIm_3_)_2_	*P*3̅1*c*	8.7338(9)	17.621(2)	1164	2

a SG represents space group and *Z* represents the number of formula units per unit cell. Additional crystallographic data are available in the Supporting Information.

The symmetry imposed by the 3-fold inversion axis, which runs through Mg1 and Mg2 atoms, makes the coordination complexes linear. The distance between the neighboring Mg atoms (Mg1–Mg2) is 8.3753(6) Å. Each Mg is octahedrally coordinated with six nitrogen atom donors from imidazole moieties with the Mg–N distances between 2.1931(11) and 2.2060(12) Å.

The adjacent Mg atoms are connected via three in situ-formed bis(imidazolyl)borate bridging anions {(Im)BH_2_(Im)}^−^ which are representatives of poly(1-imidazolyl)borate family of ligands. Terminal Mg atoms are additionally coordinated with three monodentate imidazole ligands, resulting in the formula [Mg_3_{(Im)BH_2_(Im)}_6_(ImH)_6_]·CH_3_CN.

Even though (1-imidazolyl)borates represent a well-established ligand system (over 100 hits in the CSD^29^), to our knowledge only two structures containing ligands with unsubstituted imidazolates in the moiety {(Im)BH_2_(Im)}^−^, coordinated to metal ions via the nitrogen donors, have been described until now.^30^ In the first (refcode QESTIR in the CSD^29^), the ligand is monodentate while in the second (refcode QESTUD), two bridging {(Im)BH_2_(Im)}^−^ ligands are present. In both compounds silver atoms serve as coordination centers, and the coordination spheres of the central atoms are completed by assistance from phosphine-like ligands coordinated to Ag^+^. Even though Mg-based trinuclear building units with linear geometry are commonly found in metal–organic framework structures, where adjacent Mg^2+^ centers are bridged via carboxylate ligands,^52^ bridging through hybrid organic–borohydride species is a novelty.

There are two main differences between the known linear carboxylate-based trinuclear Mg complexes and the complex reported herein. The first is due to coordination diversity of carboxylates, which can adopt multiple ligation possibilities (bidentate bridging, symmetric and asymmetric chelation, etc.) even in a single complex. In contrast, the bis(imidazolyl)borate ligand is limited to a fairly uniform and predictable bidentate bridging ligation through two free imidazolate nitrogen atoms. The second difference is the Mg–Mg distance in the complexes, which ranges roughly between 3.4 and 4.3 Å in carboxylate-based complexes and is much smaller than in the complex reported herein (8.734 Å). Thus, the three long bis(imidazolyl)borate bridges between the Mg^2+^ centers lead to the formation of characteristic cages with a diameter of 4.6 Å, possibly capable of capturing/adsorbing selected species, that have not been observed previously (Figure S2 in the Supporting Information).

Linear trinuclear coordination units with a length of ∼23 Å are packed in typical rhombohedral stacking, where the cylindrical [Mg_3_{(Im)BH_2_(Im)}_6_(ImH)_6_] moieties align parallel along the *c* direction of the unit cell (with Mg atoms positioned on the 3-fold inversion axes). Molecules of the same kind in the neighboring column are shifted by 1/3 along the *c* direction, thus bringing together the parts of the molecules capable of forming moderately strong BH···HN dihydrogen bonds between the negative hydride bonded to boron and the positive hydrogen bonded to nitrogen. These hydrogen bonds hold the trinuclear complexes together and stabilize the entire structure in a pseudo-three-dimensional array (Figure S2 in the Supporting Information). The observed BH···HN dihydrogen bonds are similar to the interactions reported in chemically related compounds LiBH_4_·NH_3_^12^ and Mg(BH_4_)_2_·NH_3_,^15^ where they facilitate structural fexibility, allowing for conduction of Li^+^ and Mg^2+^, respectively. Due to the shift of the neighboring columns along the *c* axis, voids are formed at each end of the coordination complexes, which are filled with acetonitrile solvent in the analyzed form of the compound. The acetonitrile molecule is disordered as already described in crystal structure optimizations in the Experimental Section, and its role in the stability of the crystal is discussed below.

**Figure 2:**
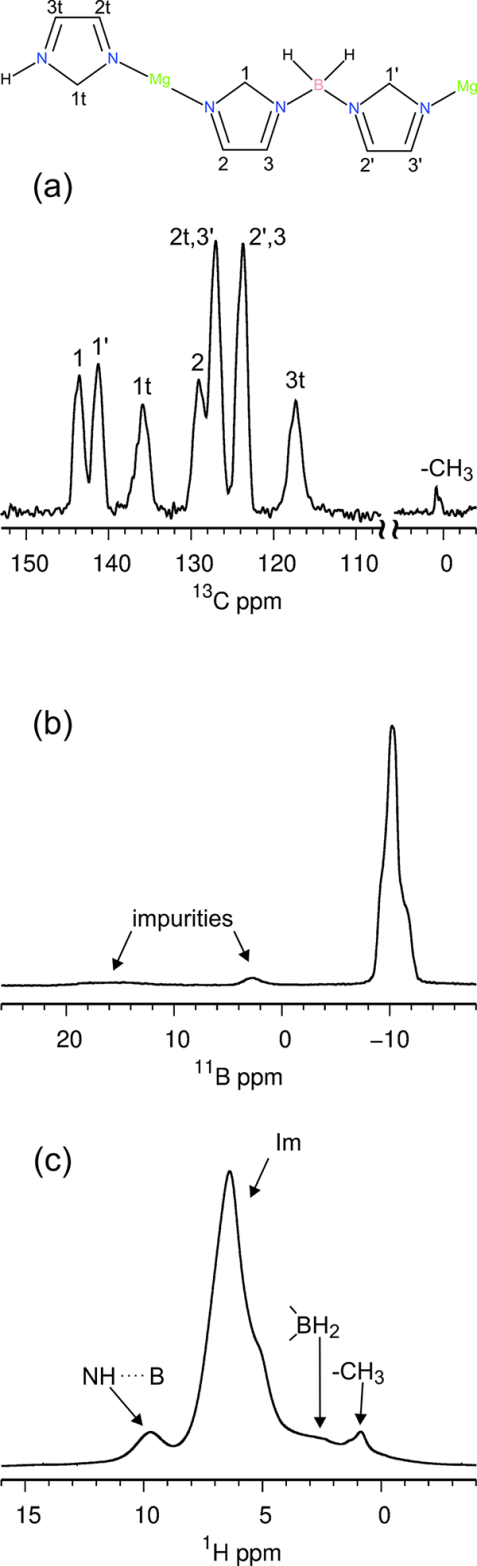
^1^H–^13^C LG-CPMAS (a), ^11^B MAS (b), and ^1^H MAS (c) NMR spectra of the title system in the form of acetonitrile solvate. Assignment of carbon signals is based on first-principles calculations of isotropic chemical shifts, whereas assignment of hydrogen signals is based on heteronuclear-correlation NMR experiments and first-principles calculations. Scheme on top offers a key for the labeling of carbon signals.

The title system in the form of acetonitrile solvate was inspected also by ^1^H, ^11^B, and ^13^C solid-state NMR spectroscopy (Figure 2). The carbon NMR spectrum is the most informative. It exhibits seven strong signals in the chemical shift range between 110 and 150 ppm and a weak signal at about 1 ppm. The strong signals belong to carbon atoms of the three crystallographically distinct Im molecules. A quantitative measurement shows that the ratio of the relative intensities of these signals is approximately 1:1:1:1:2:2:1. This means that some carbon sites have very similar local environments, which lead to overlap of the corresponding resonance lines. Detailed assignment of the resolved signals can be accomplished by DFT/GIPAW calculations of the isotropic chemical shifts, which are based on the structural model derived from the XRD analysis. The agreement between the calculated and the detected chemical shifts is quite good (Supporting Information, Figure S3). It is worth noting that the carbon signals resonating at 117 and 136 ppm are somewhat broader than the rest of the carbon NMR signals. These two signals belong to C1t and C3t atoms of the terminal, monodentatly bound Im unit. Broadening of these signals suggests that the positions (orientations) of the terminal ImH species are less well-defined than the positions of the bridging, bidentately bound Im species. The weak carbon contribution resonating at about 1 ppm belongs to methyl carbon atoms of the acetonitrile molecules. Close inspection of this contribution shows that it is composed of more than one resonance line, thus suggesting that the acetonitrile molecules can adopt different orientations within the crystals of the compound.

^11^B MAS NMR spectrum of the acetonitrile solvate exhibits one strong signal with a typical quadrupolar line shape at about −10 ppm, and two weak signals resonating at 4 ppm and between 12 and 20 ppm. Given that the structural model of the compound predicts a single boron environment and that the intensities of the latter two signals are much lower than the intensity of the signal at −10 ppm, the weak signals very likely belong to impurities, which in a powdered sample are present in a very low concentration. The quadrupolar parameters, determined by the analysis of the experimental boron NMR spectrum (*C*_Q_ = 1.4 MHz, η_Q_ = 0.73), agree very well with the parameters calculated with the DFT/PAW calculations (*C*_Q_ = 1.5 MHz, η_Q_ = 0.70); this provides additional support to the proposed structural model.

^1^H MAS NMR spectrum is the least resolved among the three NMR spectra. The most intense signal resonates at about 6.5 ppm and comprises contributions of hydrogen atoms of the three nonequivalent Im species. The assignment is clearly confirmed by the two-dimensional ^1^H–^13^C heteronuclear-correlation (HETCOR) NMR spectrum and by DFT/GIPAW calculations (Supporting Information, Figure S4). The two-dimensional spectrum also shows that the relatively sharp ^1^H NMR signal close to 1 ppm belongs to the methyl protons of the acetonitrile molecules. ^1^H–^11^B HETCOR experiment elucidates the origin of a low, broad signal extending between 2 and 4 ppm (Supporting Information, Figure S4); this signal belongs to hydrogen atoms attached to boron. The most difficult is the assignment of the well resolved signal at ∼9.7 ppm, as it does not exhibit any correlation peak in the above-described HETCOR spectra. Its chemical shift suggests that the signal could belong to protons in a moderately strong hydrogen bond. According to the proposed structural model, BH···HN dihydrogen bonds exist between the N–H atoms of the terminal ImH species and the borohydride groups. Indeed, the DFT/GIPAW calculations predict the isotropic chemical shift of 10 ppm for these hydrogen nuclei, which again agrees very well with the observation.

From the results of geometry optimizations of the acetonitrile solvate crystal structure with three different functionals (Table S4 in the Supporting Information), it is apparent that functional RP shows the best agreement with experimental volume (discrepancy of 0.3%). Unit cell dimensions of the optimized structure changed anisotropically relative to experimental data with parameter *a* changing 0.02% and parameter *c* 0.29%. This suggests that the structure is most sensitive to interaction changes in the direction of axis *c*. The acetonitrile molecule is involved in many short contacts with neighboring molecules of [Mg_3_{(Im)BH_2_(Im)}_6_(ImH)_6_]. Predominant are of the type BH···HC, where one methyl group bridges two BH_2_ groups from different [Mg_3_{(Im)BH_2_(Im)}_6_(ImH)_6_] complexes, thus providing additional cohesion between the main building units in the structure. The H···H distances calculated by DFT in two identified BH···HC interactions are 2.13 and 2.17 Å, which is shorter than the sum of van der Waals radii of 2.40 Å and can be regarded as a type of weak dihydrogen bond^53,54^ where the HC moiety is bonded to an electron-attracting group (in this case CN), making the corresponding hydrogen slightly positive (Figure S3 in the Supporting Information). Furthermore, dispersion interactions appear to importantly stabilize the structure, because when dispersion corrections are omitted, the optimized unit cell parameters increase dramatically (by more than 10%; see Table S4 in the Supporting Information). Otherwise, regardless of the applied computational model, the agreement between computed and crystallographically determined atomic positions is very good, the computed interatomic distances and angles differing by less than 1% from the respective experimental values (see Table S2 in the Supporting Information).

Optimizations of model structures with six different acetonitrile orientations resulted in similar structure energies. The difference between the highest and the lowest energy was 0.023 eV (2.2 kJ/mol) per one acetonitrile molecule, suggesting that the molecules are able to rotate around the 3-fold inversion axis virtually freely, because the energy differences are comparable to the thermal motion energy (*k*_B_*T*) at room temperature. The structure with the most stable orientation of acetonitrile molecules was used in further calculations. Optimized atomic coordinates of an acetonitrile molecule in the optimized structure with the lowest energy were used in structure refinement process as described in the Experimental Section, Single Crystal X-ray Diffraction, since DFT methods provided more accurate information than diffraction due to crystallographic disorder.

Interaction energy of acetonitrile with coordination complexes, estimated by subtracting the energy of an acetonitrile molecule in vacuum (multiplied by the number of acetonitrile molecules in the unit cell) and the energy of a model structure without acetonitrile molecules from the energy of the crystallography-predicted structure with the lowest energy was −0.422 eV (−40.7 kJ/mol), normalized to a single acetonitrile molecule. Considering negative interaction energy and the increase in optimized unit cell volume upon removal of acetonitrile, it can be concluded that acetonitrile stabilizes the structure with moderately strong polar interactions. In contrast to the observed difference of accuracy/sensitivity of the employed DFT approach between crystallographic axes, the volume increase upon removal of the acetonitrile molecule from the already optimized structure appears to be quite isotropic, because all of the unit cell vectors elongate by a comparable amount of 0.3–0.4%. This suggests that the acetonitrile molecule stabilizes the structure almost equally in all spatial directions.

### Stability, Purity, and Sorption Properties of the Acetonitrile Solvate

**Figure 3:**
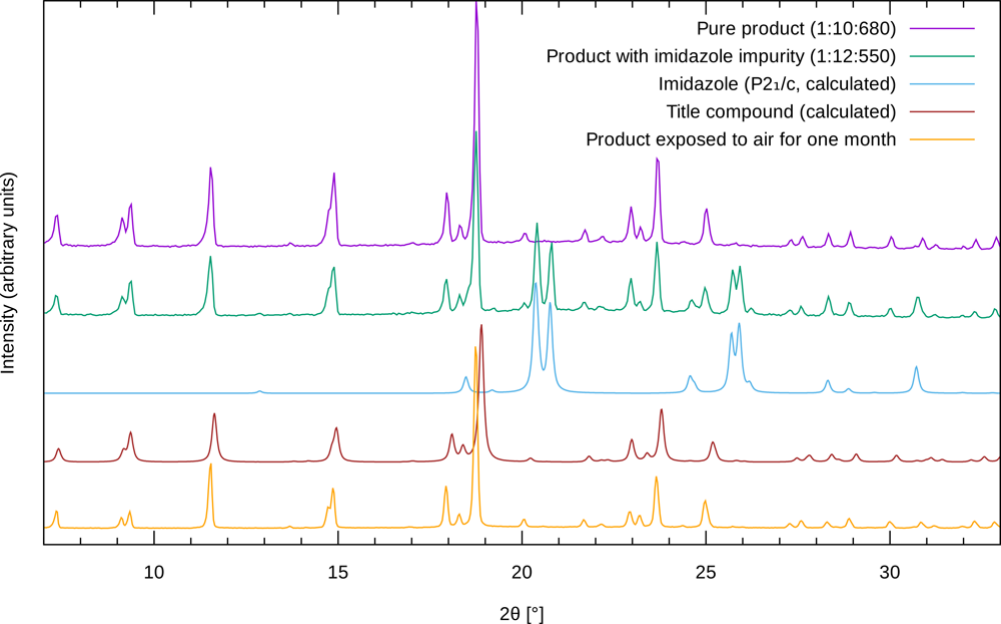
Powder X-ray patterns of pure and impure products, obtained by the solvothermal method, and product exposed to air for 1 month compared to calculated patterns of imidazole and the title compound in the form of acetonitrile solvate. Some patterns are scaled for ease of comparison. A slight mismatch between positions of measured and calculated peaks of the title compound is a consequence of different measurement temperatures. Molar ratios of reactants used to obtain the products are given in parentheses in the following order: Mg(BH_4_)_2_, ImH, CH_3_CN.

Powder X-ray patterns of all solvothermally obtained products and their single-crystal and powder fractions contained a crystalline phase with the same structure as determined by single-crystal diffraction, while some samples also contained an imidazole impurity (Figure 3). An imidazole polymorph with space group *P*2_1_/*c* was sometimes observed in the powder phase of the samples. Powder patterns of crystals, formed on the surface of the liner, generally exhibited higher intensity peaks than patterns of the powder phase.

**Figure 4:**
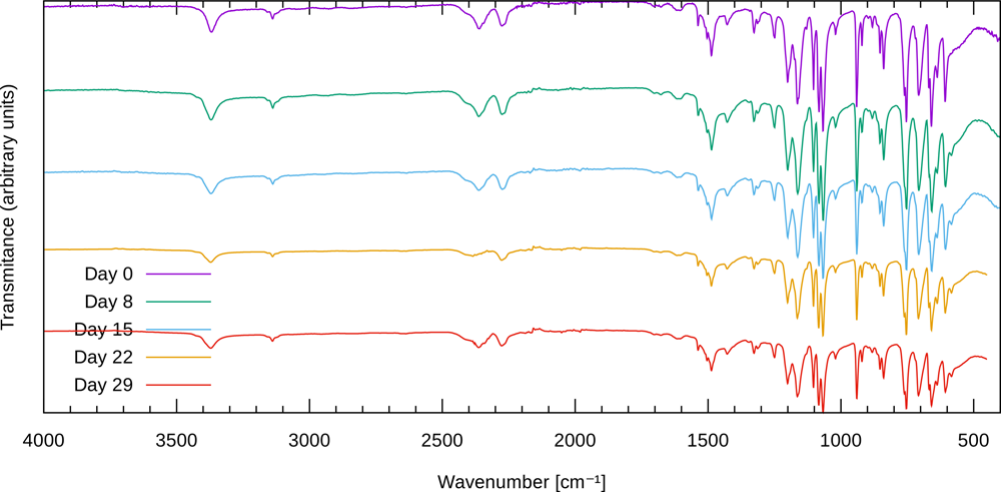
Periodically recorded infrared spectra of a powdered sample of the acetonitrile solvate exposed to air.

An XRD scan of the acetonitrile solvate sample exposed to air confirmed that the compound is stable in air for at least 1 month at room conditions. This is in agreement with periodically recorded infrared spectra as shown in Figure 4, which differ minimally from one another and indicate identical composition of the sample across the whole sampling timeline. Differences between the spectra are attributed to different sample loadings and applied pressures during data acquisition, because they mostly manifest in the form of slightly different peak intensities.

**Figure 5:**
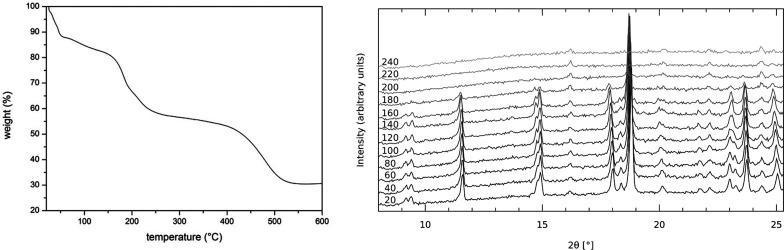
Thermogravimetric curve (left) and temperature-programmed XRD powder patterns (right) of the acetonitrile solvate. The temperature (°C) is labeled on each powder pattern.

Even though [Mg_3_{(Im)BH_2_(Im)}_6_(ImH)_6_]·CH_3_CN is defined as the molecular structure, the described neighboring moieties are stacked through BH···HN dihydrogen bonds into pseudo-three-dimensional structure. Thermogravimetric analysis along with the temperature-programmed powder XRD shown in Figure 5 demonstrates the thermal behavior of the overall structure. The thermogravimetric curve basically shows four distinct weight losses in different temperature regimes. The first loss below 60 °C can be assigned to the removal of the acetonitrile solvent located on the material’s surface. A rapid drying process seen in the first step is followed by more gradual removal of acetonitrile involved in the material’s crystal structure in the temperature region from 60 °C to 150 °C. The molecular structure and crystal packing seem to withstand the removal of solvent as indicated by the XRD patterns measured up to 160 °C. The remaining two steps with similar weight contributions between 160 °C and 500 °C are most probably due to the decomposition of protonated (terminal) and deprotonated (bridging) ligands together with the removal of BH_2_ groups. Removal of ligand fragments is accompanied by substantial amorphization of material.

As described above, the trinuclear molecular moieties form cages with a diameter of 4.6 Å and can therefore potentially adsorb small gas molecules. Nevertheless, N_2_ sorption isotherms measured at 77 K show that these cages are not accessible for N_2_ molecules. The material exhibits type III isotherm typical for nonporous adsorbents with negligible BET surface area of 9 m^2^/g. The rapid increase of N_2_ uptake occurs only above relative pressures of 0.8 due to the N_2_ condensation on the surface of the material. A similar effect can be observed for H_2_ adsorption at 77 K with a gradual near-linear increase of uptake up to 10 bar with no sign of saturation. The orientation of imidazolate ligands that form cages prevents the access of even the smallest gas molecules to adsorb within the trinuclear moieties. N_2_ and H_2_ isotherms are shown in Figure S8 in the Supporting Information.

### Characterization of the Imidazole Solvate

**Figure 6:**
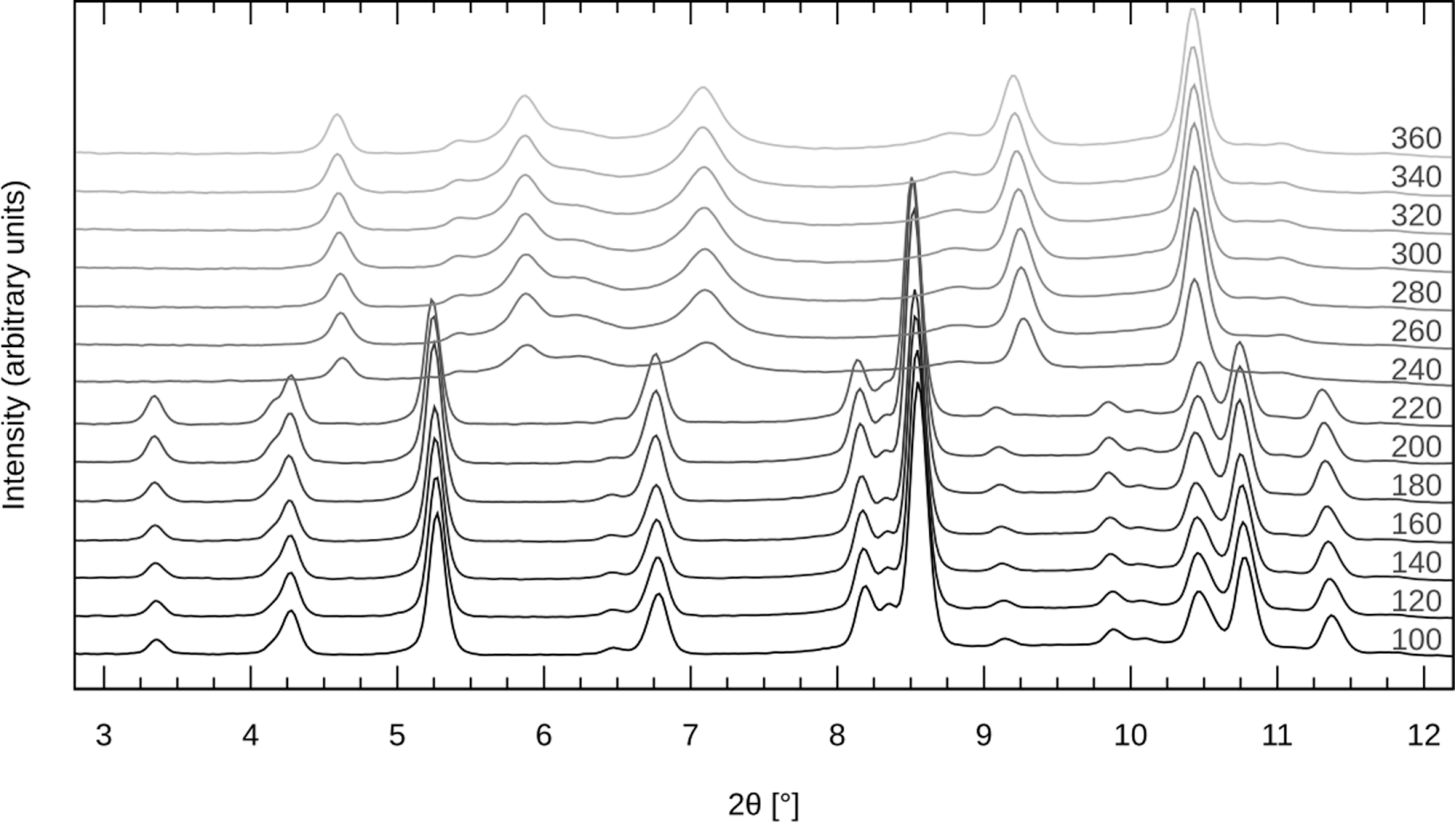
Synchrotron temperature-programmed XRD powder patterns of the imidazole solvate. The temperature (°C) is labeled on each powder pattern.

Temperature-programmed X-ray powder diffraction shows that the room temperature powder pattern of the title compound, prepared from the imidazole melt, is very similar to that of the acetonitrile solvate (Figure 6), confirming the structural similarity. In contrast to the acetonitrile solvate, however, the imidazole solvate is stable up to 220 °C and transforms to another crystalline product upon thermal decomposition. The product is stable up to 360 °C. Conversely, acetonitrile solvate decomposes to an amorphous material at temperatures above 160 °C.

The material, synthesized in the imidazole melt, was expected to be a rather pure solvent-free form of the title compound. Its powder pattern was completely indexable by a rhombohedral unit cell (Table 1) very similar to that of the acetonitrile solvate, and its ^11^B NMR spectrum in D_2_O (see the Supporting Information) revealed only bisubstituted borohydride groups (BH_2_) and a small amount of BH_4_ impurity from Mg(BH_4_)_2_. However, it was proven by Rietveld refinement that this material does not consist of exclusively trinuclear [Mg_3_{(Im)BH_2_(Im)}_6_(ImH)_6_] moieties. From the difference Fourier map it was evident that there is missing electron density between these moieties (the space occupied by acetonitrile molecules in the acetonitrile solvate; see the Supporting Information).

By occupying the aforementioned space with the model of an imidazole molecule, a very good Rietveld fit was achieved. The population parameter of imidazole was refined to 0.159, which is very close to the maximum possible of 1/6 in regards to the 6-fold symmetry of the site, suggesting that the voids are nearly fully occupied. Similar refinements were performed with patterns collected at 80 °C (see the Supporting Information), 160 °C, 200 °C, and 220 °C and resulted in imidazole population parameters of 0.155, 0.154, 0.136, and 0.114, respectively, indicating that the voids are still 68% occupied at the highest temperature, just before the structure disintegrates.

**Figure 7:**
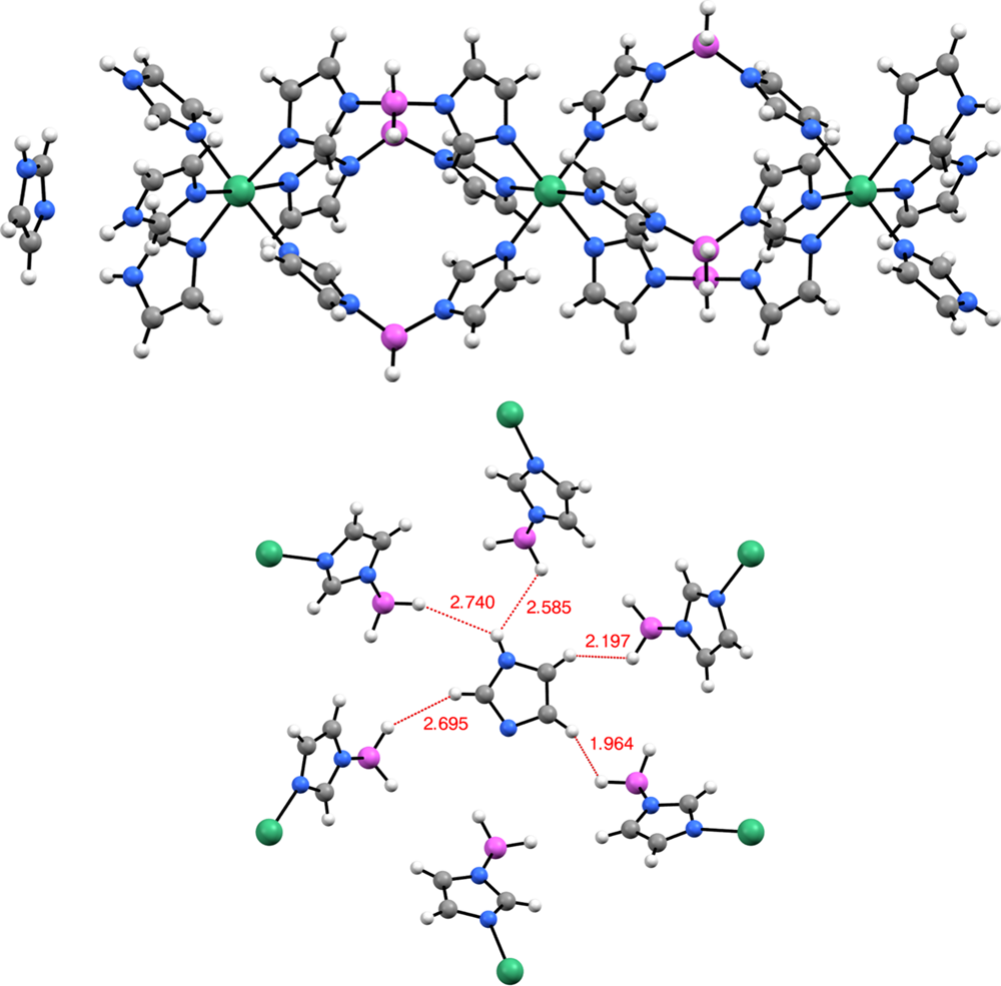
Structural model of [Mg_3_{(Im)BH_2_(Im)}_6_(ImH)_6_]·ImH (top) and interactions of the imidazole solvent molecule with nearby BH_2_ groups of the trinuclear moieties (bottom).

These findings confirm that formation and stability of the rhombohedral structure, built principally from [Mg_3_{(Im)BH_2_(Im)}_6_(ImH)_6_] moieties, is solvent-dependent. On one hand, the solvent can be viewed as a spacer or void filler, fitting into the void between the trinuclear moieties, when they arrange in the rhombohedral manner so that BH_2_ groups of one unit are close to NH groups of the neighboring ones to maintain BH···HN dihydrogen bonds. On the other hand, the solvent molecule is also involved in interactions with BH_2_ groups of the surrounding molecules (Figure 7). This seems to apply for both solvents studied and was also confirmed by DFT calculations for the case of acetonitrile.

### Crystal Structure of Mg(BHIm_3_)_2_

**Figure 8:**
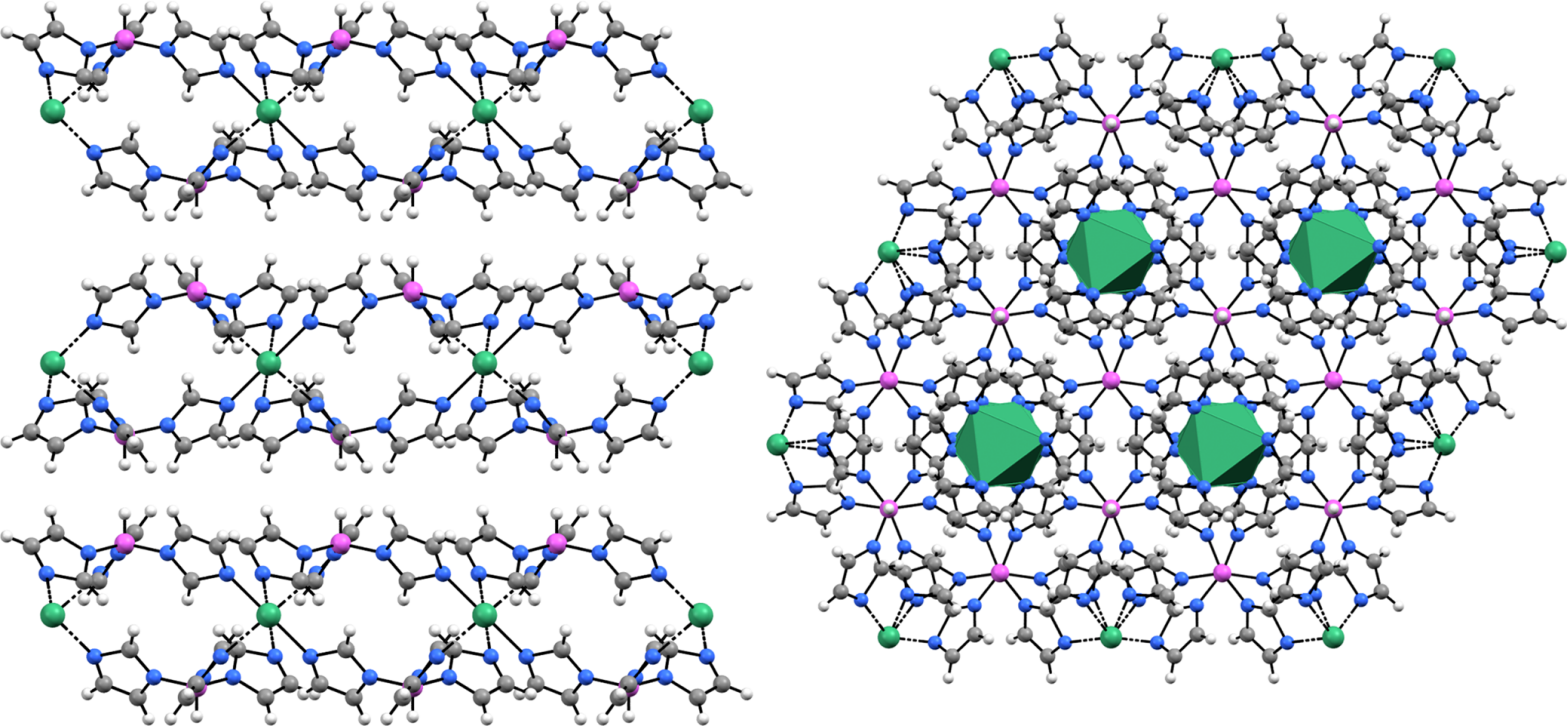
Packing of Mg(BHIm_3_)_2_ layers (left) and a view of the same structure in the direction of layer stacking (right).

The crystalline phase with molecular formula C_18_H_20_B_2_MgN_12_, formed by thermal decomposition of the imidazole solvate, generally exhibited broad and less reproducible peaks in powder XRD patterns. Despite that, omitting some inconsistent low intensity peaks, we found a trigonal unit cell (Table 1) using Dicvol.^55^ Based on the shape and possible symmetries of the unit cell, the chemical composition of the imidazole solvate structure, high-temperature conditions, and structure density considerations, multiple possible structural models were constructed. Among those were models containing BHIm_3_ moieties, assuming a reaction between hydrogen atoms of terminal imidazole molecules of trinuclear moieties and hydrides of BH_2_ bridges. Because there are several known structures containing the BHIm_3_ moiety in the CSD, it was possible to construct a realistic model for such a structural fragment. Upon Rietveld refinement, one of the model structures readily converged to a reasonable arrangement (Figure 8) with an acceptable fit of the measured powder pattern. A detailed description of the structure solution process and the Rietveld fit of the measured powder pattern are available in the Supporting Information.

The structure consists of layers, having the formula Mg(BHIm_3_)_2_, where Mg atoms are sandwiched between the BHIm_3_ moieties so that the nitrogen imidazolate atoms, not bound to boron, form a nearly regular octahedron around Mg. The layers are stacked in a primitive trigonal manner, and there are no remarkable short contacts between the layers (Figure 8). More data can be found in the Supporting Information.

## Conclusion

With the aim of preparing compounds consisting of metal coordination centers, imidazolate linkers, and borohydride units, we synthesized acetonitrile and imidazole solvates of the trinuclear linear magnesium complex [Mg_3_{(Im)BH_2_(Im)}_6_(ImH)_6_]. In both cases, the bidentate bis(imidazolyl)borate ligands {(Im)BH_2_(Im)}^−^ were formed in situ from borohydride and imidazole with the release of hydrogen.

The trinuclear complex reported here for the first time was identical in both syntheses. It differs from known linear carboxylate-based trinuclear magnesium complexes by its size (it is about twice as long) and by the presence of two cavities between the magnesium centers, each about 4.6 Å in diameter. Sorption measurements revealed that the cavities between the bis(imidazolyl)borate bridges are inaccessible to even the smallest molecules, such as H_2_.

Detailed structural characterization of the acetonitrile solvate by single-crystal X-ray diffraction, solid-state NMR, and DFT calculations revealed that packing is directed by the BH···HN dihydrogen bonds that hold the complexes together in the lateral directions. Similar interactions have been described in fast Mg^2+^ ionic conductors. The acetonitrile molecules fill the cavities between the complexes in the longitudinal direction and additionally stabilize the structure via BH···HC interactions.

The acetonitrile solvate is stable at temperatures up to 160 °C, whereas the imidazole solvate transforms into a new crystalline phase upon heating above 220 °C. The new phase consists of trisubstituted borohydrides forming two-dimensional layers with the formula Mg(BHIm_3_)_2_. Its crystal structure confirms that two different types of reactive hydrogen atoms (hydride bonded to boron and hydrogen bonded to nitrogen) can comproportionate to form new compounds.

The investigation described here has clearly demonstrated that the integration of light metal-based imidazolates coordinated via N atoms and boron hydrides, utilizing the BH_2_ bridging, leads to reactive precursors in which the reactive hydride atoms can be used for further transformations. These may lead to the development of a new generation of hybrid materials for energy storage and possibly other applications.

## Supporting Information Available

Detailed synthesis procedure, additional crystallographic, NMR, sorption, and DFT data, detailed description of Rietveld refinements, and additional figures of the crystal strutures (PDF)

## References

[ref1] Díaz U.; Brunel D.; Corma A. Catalysis using multifunctional organosiliceous hybrid materials. Chem. Soc. Rev. 2013, 42, 4083–4097. 10.1039/c2cs35385g. 23288312

[ref2] Lee J.; Farha O. K.; Roberts J.; Scheidt K. A.; Nguyen S. T.; Hupp J. T. Metal–organic framework materials as catalysts. Chem. Soc. Rev. 2009, 38, 1450–1459. 10.1039/b807080f. 19384447

[ref3] Hoffmann F.; Cornelius M.; Morell J.; Fröba M. Silica-Based Mesoporous Organic–Inorganic Hybrid Materials. Angew. Chemie Int. Ed. 2006, 45, 3216–3251. 10.1002/anie.200503075. 16676373

[ref4] Taguchi A.; Schüth F. Ordered mesoporous materials in catalysis. Microporous Mesoporous Mater. 2005, 77, 1–45. 10.1016/j.micromeso.2004.06.030.

[ref5] Centi G.; Perathoner S. Catalysis by layered materials: A review. Microporous Mesoporous Mater. 2008, 107, 3–15. 10.1016/j.micromeso.2007.03.011.

[ref6] Taylor-Pashow K. M. L.; Della Rocca J.; Huxford R. C.; Lin W. Hybrid nanomaterials for biomedical applications. Chem. Commun. 2010, 46, 5832–5849. 10.1039/c002073g. 20623072

[ref7] Li Z.; Barnes J. C.; Bosoy A.; Stoddart J. F.; Zink J. I. Mesoporous silica nanoparticles in biomedical applications. Chem. Soc. Rev. 2012, 41, 2590–2605. 10.1039/c1cs15246g. 22216418

[ref8] Cobo I.; Li M.; Sumerlin B. S.; Perrier S. Smart hybrid materials by conjugation of responsive polymers to biomacromolecules. Nat. Mater. 2015, 14, 143–159. 10.1038/nmat4106. 25401924

[ref9] Owens G. J.; Singh R. K.; Foroutan F.; Alqaysi M.; Han C.-M.; Mahapatra C.; Kim H.-W.; Knowles J. C. Sol–gel based materials for biomedical applications. Prog. Mater. Sci. 2016, 77, 1–79. 10.1016/j.pmatsci.2015.12.001.

[ref10] Li J.; Zhang J. Z. Optical properties and applications of hybrid semiconductor nanomaterials. Coord. Chem. Rev. 2009, 253, 3015–3041. 10.1016/j.ccr.2009.07.017.

[ref11] Choi H.; Yoon H. Nanostructured Electrode Materials for Electrochemical Capacitor Applications. Nanomaterials 2015, 5, 906–936. 10.3390/nano5020906. 28347044 PMC5312909

[ref12] Yan Y.; Grinderslev J. B.; Lee Y.-S.; Jørgensen M.; Cho Y. W.; Černý R.; Jensen T. R. Ammonia-assisted fast Li-ion conductivity in a new hemiammine lithium borohydride, LiBH_4_·1/2NH_3_. Chem. Commun. 2020, 56, 3971–3974. 10.1039/C9CC09990E. 32152608

[ref13] Grinderslev J. B.; Skov L. N.; Andreasen J. G.; Ghorwal S.; Skibsted J.; Jensen T. R. Methylamine Lithium Borohydride as Electrolyte for All-Solid-State Batteries. Angew. Chem., Int. Ed. 2022, e202203484. 10.1002/anie.202203484 PMC9400857 35662368

[ref14] Yan Y.; Grinderslev J. B.; Jørgensen M.; Skov L. N.; Skibsted J. r.; Jensen T. R. Ammine Magnesium Borohydride Nanocomposites for All-Solid-State Magnesium Batteries. ACS Appl. Energy Mater. 2020, 3, 9264–9270. 10.1021/acsaem.0c01599.

[ref15] Yan Y.; Dononelli W.; Jørgensen M.; Grinderslev J. B.; Lee Y.-S.; Cho Y. W.; Černý R.; Hammer B.; Jensen T. R. The mechanism of Mg^2+^ conduction in amminemagnesium borohydride promoted by a neutral molecule. Phys. Chem. Chem. Phys. 2020, 22, 9204–9209. 10.1039/D0CP00158A. 32232248

[ref16] Wang S.; Kang Y.; Wang L.; Zhang H.; Wang Y.; Wang Y. Organic/inorganic hybrid sensors: A review. Sensors Actuators B Chem. 2013, 182, 467–481. 10.1016/j.snb.2013.03.042.

[ref17] Pardo R.; Zayat M.; Levy D. Photochromic organic–inorganic hybrid materials. Chem. Soc. Rev. 2011, 40, 672–687. 10.1039/c0cs00065e. 21229130

[ref18] Pomerantseva E.; Bonaccorso F.; Feng X.; Cui Y.; Gogotsi Y. Energy storage: The future enabled by nanomaterials. Science 2019, 366, eaan8285 10.1126/science.aan8285. 31753970

[ref19] Khin M. M.; Nair A. S.; Babu V. J.; Murugan R.; Ramakrishna S. A review on nanomaterials for environmental remediation. Energy Environ. Sci. 2012, 5, 8075–8109. 10.1039/c2ee21818f.

[ref20] Mendoza-Sánchez B.; Gogotsi Y. Synthesis of Two-Dimensional Materials for Capacitive Energy Storage. Adv. Mater. 2016, 28, 6104–6135. 10.1002/adma.201506133. 27254831

[ref21] Hirscher M.; Yartys V. A.; Baricco M.; Bellosta von Colbe J.; Blanchard D.; Bowman R. C.; Broom D. P.; Buckley C. E.; Chang F.; Chen P.; et al. Materials for hydrogen-based energy storage – past, recent progress and future outlook. J. Alloy. Compd. 2020, 827, 153548 10.1016/j.jallcom.2019.153548.

[ref22] Kadota K.; Duong N. T.; Nishiyama Y.; Sivaniah E.; Kitagawa S.; Horike S. Borohydride-containing coordination polymers: synthesis, air stability and dehydrogenation. Chem. Sci. 2019, 10, 6193–6198. 10.1039/C9SC00731H. 31360426 PMC6585883

[ref23] Rossin A.; Tuci G.; Luconi L.; Giambastiani G. Metal–Organic Frameworks as Heterogeneous Catalysts in Hydrogen Production from Lightweight Inorganic Hydrides. ACS Catal. 2017, 7, 5035–5045. 10.1021/acscatal.7b01495.

[ref24] Sun W.; Li S.; Mao J.; Guo Z.; Liu H.; Dou S.; Yu X. Nanoconfinement of lithium borohydride in Cu-MOFs towards low temperature dehydrogenation. Dalton Trans. 2011, 40, 5673–5676. 10.1039/c0dt01727b. 21505678

[ref25] Bhakta R. K.; Herberg J. L.; Jacobs B.; Highley A.; Behrens R.; Ockwig N. W.; Greathouse J. A.; Allendorf M. D. Metal–Organic Frameworks As Templates for Nanoscale NaAlH_4_. J. Am. Chem. Soc. 2009, 131, 13198–13199. 10.1021/ja904431x. 19719170

[ref26] Andrew Lin K.-Y.; Chen S.-Y. Bromate reduction in water by catalytic hydrogenation using metal–organic frameworks and sodium borohydride. RSC Adv. 2015, 5, 43885–43896. 10.1039/C5RA05705A.

[ref27] Ingleson M.; Barrio J.; Bacsa J.; Steiner A.; Darling G.; Jones J.; Khimyak Y.; Rosseinsky M. Magnesium Borohydride Confined in a Metal–Organic Framework: A Preorganized System for Facile Arene Hydroboration. Angew. Chem., Int. Ed. 2009, 48, 2012–2016. 10.1002/anie.200804196. 19180619

[ref28] Zhang H.-X.; Liu M.; Wen T.; Zhang J. Synthetic design of functional boron imidazolate frameworks. Coordin. Chem. Rev. 2016, 307, 255–266. 10.1016/j.ccr.2015.08.003.

[ref29] Groom C. R.; Bruno I. J.; Lightfoot M. P.; Ward S. C. The Cambridge Structural Database. Acta Crystallogr. 2016, B72, 171–179. 10.1107/S2052520616003954 PMC4822653 27048719

[ref30] Effendy; Gioia Lobbia G.; Pellei M.; Pettinari C.; Santini C.; Skelton B. W.; White A. H. Bridged poly(1-imidazolyl)borate silver(I) complexes containing tertiary mono(phosphine) ligands. The first structurally authenticated bis(imidazolyl)borate metal complex. J. Chem. Soc., Dalton Trans. 2001, 528–534.

[ref31] Shuto Y.; Yamamura T.; Tanaka S.; Yoshimura M.; Kitamura M. Asymmetric NaBH4 1,4-Reduction of C3-Disubstituted 2-Propenoates Catalyzed by a Diamidine Cobalt Complex. ChemCatChem. 2015, 7, 1547–1550. 10.1002/cctc.201500260.

[ref32] Leiner S.; Mayer P.; Nöth H. Synthesis and Structures of LiBH_4_ Complexes with N-Heterocycles. Z. Naturforsch. 2009, 64b, 793–799. 10.1515/znb-2009-0703.

[ref33] Morelle F. Hybrid hydridic frameworks by the combination of complex hydrides and nitrogen-based organic ligands. Ph.D. thesis, Université Catholique de Louvain, Louvain-la-Neuve, Belgium, 2017.

[ref34] Skripov A. V.; Dimitrievska M.; Babanova O. A.; Skoryunov R. V.; Soloninin A. V.; Morelle F.; Filinchuk Y.; Faraone A.; Wu H.; Zhou W.; et al. Low-Temperature Rotational Tunneling of Tetrahydroborate Anions in Lithium Benzimidazolate-Borohydride Li_2_(bIm)BH_4_. J. Phys. Chem. C 2019, 123, 20789–20799. 10.1021/acs.jpcc.9b06083. PMC7526645 33005285

[ref35] CrysAlisPro. Agilent Technologies Ltd, Yarnton, Oxfordshire, England, 2014.

[ref36] Palatinus L.; Chapuis G. SUPERFLIP – a computer program for the solution of crystal structures by charge ipping in arbitrary dimensions. J. Appl. Crystallogr. 2007, 40, 786–790. 10.1107/S0021889807029238.

[ref37] Sheldrick G. M. Crystal structure refinement with SHELXL. Acta Crystallogr. 2015, C71, 3–8. 10.1107/S2053229614024218 PMC4294323 25567568

[ref38] Macrae C. F.; Sovago I.; Cottrell S. J.; Galek P. T. A.; McCabe P.; Pidcock E.; Platings M.; Shields G. P.; Stevens J. S.; Towler M.; et al. *Mercury 4.0*: from visualization to analysis, design and prediction. J. Appl. Crystallogr. 2020, 53, 226–235. 10.1107/S1600576719014092. 32047413 PMC6998782

[ref39] Kresse G.; Hafner J. Ab initio molecular dynamics for liquid metals. Phys. Rev. B 1993, 47, 558–561. 10.1103/PhysRevB.47.558. 10004490

[ref40] Kresse G.; Furthmüller J. Efficiency of ab-initio total energy calculations for metals and semiconductors using a plane-wave basis set. Comput. Mater. Sci. 1996, 6, 15–50. 10.1016/0927-0256(96)00008-0. 9984901

[ref41] Kresse G.; Furthmüller J. Efficient iterative schemes for ab initio total-energy calculations using a plane-wave basis set. Phys. Rev. B 1996, 54, 11169–11186. 10.1103/PhysRevB.54.11169. 9984901

[ref42] Kresse G.; Hafner J. Norm-conserving and ultrasoft pseudopotentials for first-row and transition elements. J. Phys.: Condens. Matter 1994, 6, 8245–8257.

[ref43] Hammer B.; Hansen L. B.; Nørskov J. K. Improved adsorption energetics within density-functional theory using revised Perdew-Burke-Ernzerhof functionals. Phys. Rev. B 1999, 59, 7413–7421. 10.1103/PhysRevB.59.7413.

[ref44] Perdew J. P.; Burke K.; Ernzerhof M. Generalized Gradient Approximation Made Simple. Phys. Rev. Lett. 1996, 77, 3865–3868. 10.1103/PhysRevLett.77.3865. 10062328

[ref45] Grimme S.; Antony J.; Ehrlich S.; Krieg H. A consistent and accurate *ab initio* parametrization of density functional dispersion correction (DFT-D) for the 94 elements H-Pu. J. Chem. Phys. 2010, 132, 154104 10.1063/1.3382344. 20423165

[ref46] Kresse G.; Joubert D. From ultrasoft pseudopotentials to the projector augmented-wave method. Phys. Rev. B 1999, 59, 1758–1775. 10.1103/PhysRevB.59.1758.

[ref47] Blöchl P. E. Projector augmented-wave method. Phys. Rev. B 1994, 50, 17953–17979. 10.1103/PhysRevB.50.17953. 9976227

[ref48] Monkhorst H. J.; Pack J. D. Special points for Brillouin-zone integrations. Phys. Rev. B 1976, 13, 5188–5192. 10.1103/PhysRevB.13.5188.

[ref49] Björkman T. CIF2Cell: Generating geometries for electronic structure programs. Comput. Phys. Commun. 2011, 182, 1183–1186. 10.1016/j.cpc.2011.01.013.

[ref50] Csonka G. I.; Perdew J. P.; Ruzsinszky A.; Philipsen P. H. T.; Lebègue S.; Paier J.; Vydrov O. A.; Ángyán J. G. Assessing the performance of recent density functionals for bulk solids. Phys. Rev. B 2009, 79, 155107 10.1103/PhysRevB.79.155107.

[ref51] Coelho A. A. TOPAS and TOPAS-Academic: an optimization program integrating computer algebra and crystallographic objects written in C++. J. Appl. Crystallogr. 2018, 51, 210–218. 10.1107/S1600576718000183.

[ref52] Mazaj M.; Kasunič M.; Kaučič V.; Logar Zabukovec N. New Mg-based 4,4′-Biphenyldicarboxylate Coordination Polymer with Layered Crystal Structure. Acta Chim. Slov. 2014, 61, 432–438. 25286197

[ref53] Li J.; Zhao F.; Jing F. B–H^δ−^ σ bond as dihydrogen bond acceptor: Some theoretical observations and predictions. J. Chem. Phys. 2002, 116, 25–32. 10.1063/1.1423332.

[ref54] Robertson K. N.; Knop O.; Cameron T. S. C–H···H–C interactions in organoammonium tetraphenylborates: another look at dihydrogen bonds. Can. J. Chem. 2003, 81, 727–743. 10.1139/v03-080.

[ref55] Boultif A.; Louër D. Indexing of powder diffraction patterns for low-symmetry lattices by the successive dichotomy method. J. Appl. Crystallogr. 1991, 24, 987–993. 10.1107/S0021889891006441.

