# Supplementary material for: Trinuclear Magnesium Imidazolate Borohydride Complex
Source: Inorg Chem. 2022 Aug 2;61(32):12708–18. doi: 10.1021/acs.inorgchem.2c01319 (PMC9387531; doi:10.1021/acs.inorgchem.2c01319)
Supplement: Supplementary file 1 — ic2c01319_si_001.pdf [file ic2c01319_si_001.pdf]

Supporting Information

Trinuclear Magnesium Imidazolate Borohydride Complex

Maja Reberc,† Matjaž Mazaj,‡ Jernej Stare,‡ Marta Počkaj,† Gregor Mali,‡ Xiao Li,¶ Yaroslav Filinchuk,¶ Radovan Černý,§ and Anton Meden*,†

†*Faculty of Chemistry and Chemical Technology, University of Ljubljana, Večna pot 113, 1001 Ljubljana, Slovenia*

‡*National Institute of Chemistry, Hajdrihova 19, 1000 Ljubljana, Slovenia*

¶*IMCN Université Catholique de Louvain, Place L. Pasteur 1, B-1348, Louvain-la-Neuve, Belgium*

§*DQMP, University of Geneva, 24 quai Ernest-Ansermet, 1211 Geneva, Switzerland*

*E-mail: anton.meden@fkkt.uni-lj.si

Contents

1 Synthesis **S2**

2 Single Crystal X-ray Diffraction **S4**

3 NMR Measurements **S7**

4 Sorption Analysis **S10**

5 DFT Calculations **S11**

6 Synchrotron Powder Diffraction **S12**

6.1 $[Mg_3\{(Im)BH_2(Im)\}_6(ImH)_6] \cdot ImH$ S12

6.2 $Mg(BHIm_3)_2$ S14

1 Synthesis

$[Mg_3\{(Im)BH_2(Im)\}_6(ImH)_6] \cdot CH_3CN$ was prepared using the solvothermal synthesis method inside a glove box with nitrogen atmosphere. Magnesium borohydride ($Mg(BH_4)_2$, Sigma-Aldrich, 95 %), imidazole (ImH, Sigma-Aldrich) and acetonitrile (CH_3CN, Sigma-Aldrich, dried over CaH_2) were added in various compositions (Table S1) to a 23 mL teflon-lined stainless steel autoclave. Small bubbles were observed after the solvent was added to the solid reactants. The autoclave was heated in a standard laboratory dryer at 85 °C for 24 h. It was opened in the glove box and most of the clear solution above the solid product was removed. Remaining contents were transferred into a micro centrifuge tube and centrifuged at 5000 rpm for 5 min. The supernatant was decanted and a fine white powder was obtained and left to dry. After the remaining solvent evaporated from the teflon liner, colorless crystals formed on its surface.

Based on the results of the experiments, molar ratio of $Mg(BH_4)_2$, imidazole and acetonitrile 1:15:680 (15.2 mg, 287.5 mg and 10 mL, respectively) was chosen for production of the compound in larger quantities for characterization experiments. The decision was a compromise between product purity and yield per reaction. The reaction was carried out in parallel using four autoclaves. 259 mg of predominantly single-crystal phase, and 43 mg of powder phase were obtained. Assuming that the product is pure $[Mg_3\{(Im)BH_2(Im)\}_6(ImH)_6] \cdot CH_3CN$, the yield based on the limiting reactant $Mg(BH_4)_2$ was 55 %.

Table S1: List of solvothermal synthesis experiments performed. Quantity of reactants is given as molar ratio. Constant volume of 10 mL of CH_3CN was used for all syntheses. The chosen ratio is highlighted.

$Mg(BH_4)_2$	ImH	CH_3CN	Results
1	15	1500	Expected product
1	15	1300	Expected product
1	15	1100	Expected product
1	15	900	Expected product
1	15	680	Used to prepare samples for characterization
1	12	900	Expected product
1	12	680	Expected product
1	12	600	Expected product
1	12	550	Contains impurity imidazole ($P2_1/c$)
1	10	680	Expected product
1	8	680	Expected product

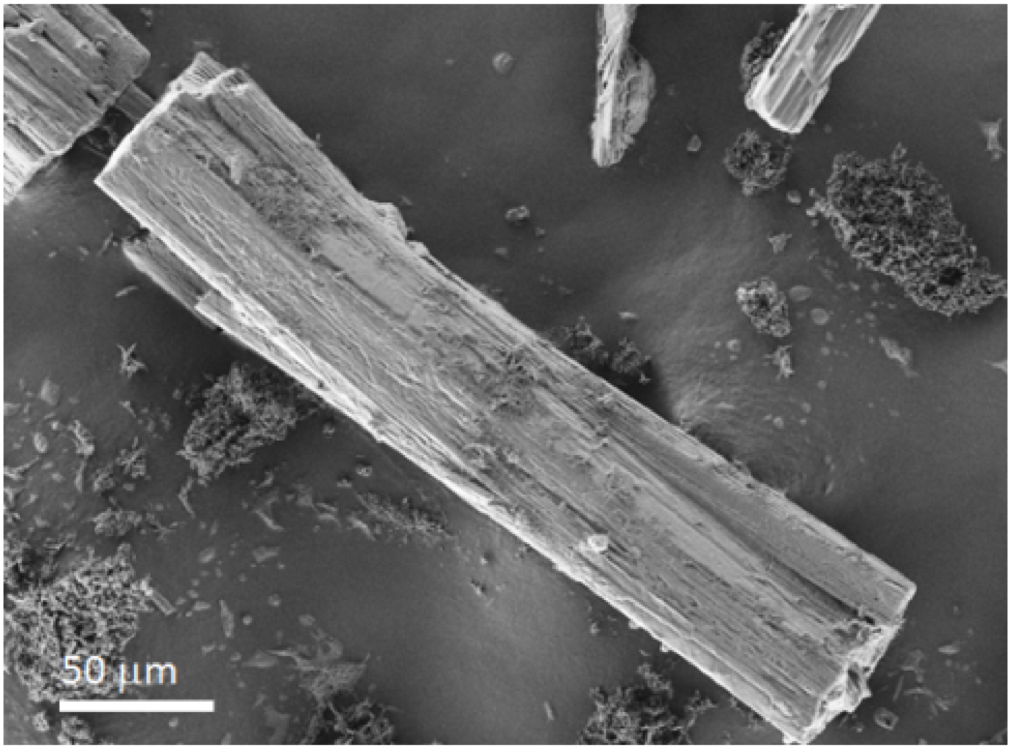

Figure S1: SEM micrograph of $[Mg_3\{(Im)BH_2(Im)\}_6(ImH)_6]\cdot CH_3CN$ crystal.

2 Single Crystal X-ray Diffraction

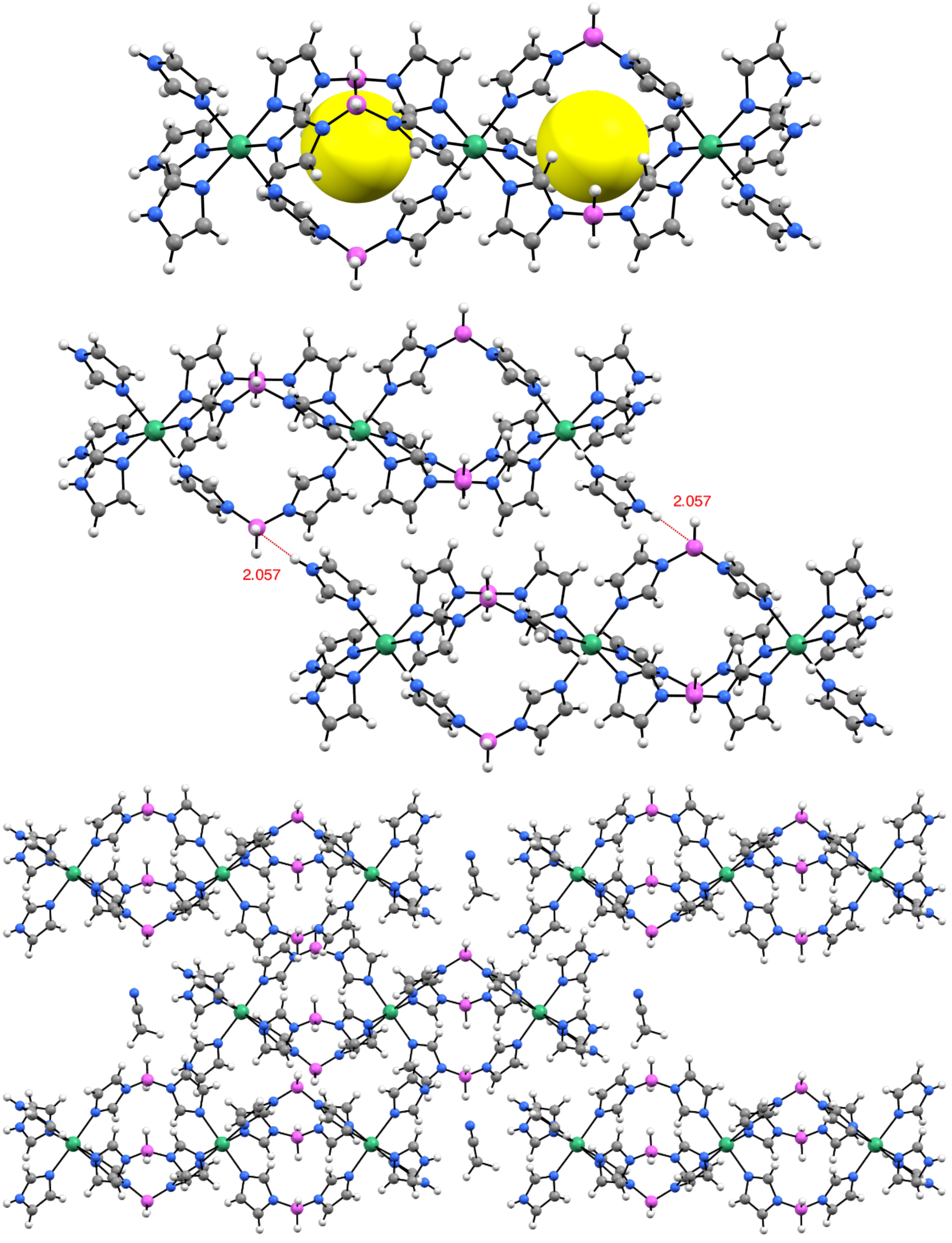

Figure S2: Above: scheme of the acetonitrile solvate showing the voids of diameter of 4.6 Å in the cages of the complex molecule as yellow spheres. Middle: two complex units interacted through B–H···H–N dihydrogen bonds (red dashed lines). Below: acetonitrile molecules located between the stacking trinuclear complex units.

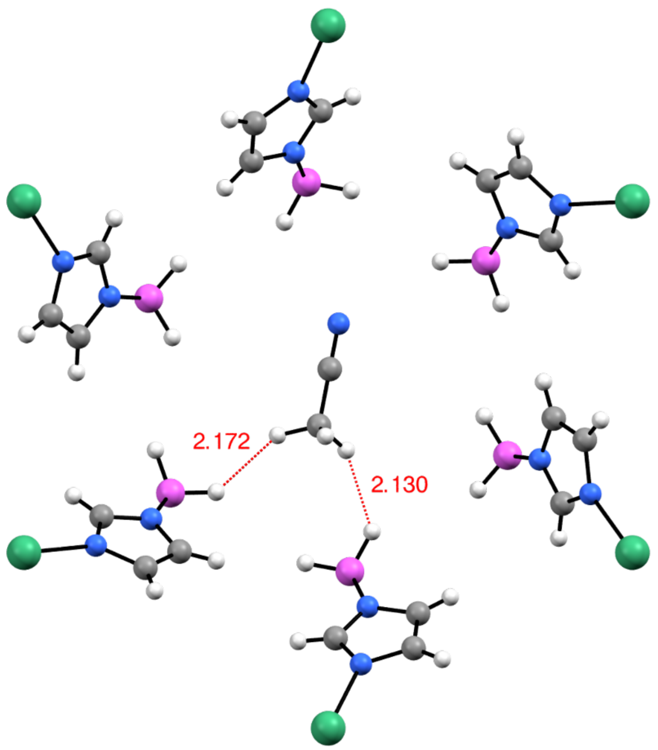

Figure S3: B–H$\cdots$H–C dihydrogen bonds between BH_2 groups of the {(Im)BH_2(Im)} ligands and CH_3 group of the acetonitrile solvent molecule. Only a single orientation of the acetonitrile molecule is shown for clarity.

Table S2: Selected bond lengths and angles (Å, °) in the experimental structure of the acetonitile solvate and average values of bond lengths and angles in the geometrically optimized structures of the compound with the lowest energy. Values in column "DFT (fixed)" were measured in unit cell constrained to experimental parameters, while column "DFT (variable)" contains values from structure with freely optimized unit cell.

**Bond or angle**	**Experimental**	**DFT (fixed)**	**DFT (variable)**
Mg1–N1	2.2060(12)	2.218	2.218
Mg1–N3	2.1931(11)	2.212	2.207
Mg2–N6	2.1952(11)	2.209	2.204
$N3^i$–Mg1–N1	177.93(5)	177.5	177.5
N3–Mg1–N1	91.62(4)	91.8	91.6
N3–Mg1–$N1^i$	89.90(4)	89.7	89.3
$N1^i$–Mg1–N1	88.04(5)	88.0	88.3
$N3^i$–Mg1–N3	90.43(4)	90.7	90.8
$N6^{ii}$–Mg2–N6	89.41(4)	89.2	89.0
$N6^i$–Mg2–N6	90.59(4)	90.8	91.0
$N6^{iii}$–Mg2–N6	180.0	179.6	179.5

Symmetry codes: (i) $-y+1$, $x-y$, z; (ii) $y+\frac{1}{3}$, $-x+y+\frac{2}{3}$, $-z+\frac{5}{3}$; (iii) $-x+\frac{4}{3}$, $-y+\frac{2}{3}$, $-z+\frac{5}{3}$.

Table S3: Crystal data, data collection and refinement of $[Mg_3\{(Im)BH_2(Im)\}_6(ImH)_6] \cdot CH_3CN$.

**Crystal data**	
Formula	$C_{56}H_{75}B_6Mg_3N_{37}$
M_r	1404.32
Cell setting, space group	Trigonal, R$\bar{3}$
a (Å)	15.1942(2)
c (Å)	28.3157(3)
V (Å^3)	5661.26(16)
Z	3
D_x (Mg m^{-3})	1.236
μ (mm^{-1})	0.878
$F(000)$	2208
Crystal form, color	prism, colorless
Crystal size (mm)	0.32 × 0.30 × 0.25
**Data collection**	
T (K)	150(2)
Radiation type, wavelength	Cu Kα, 1.541 84 Å
Diffractometer	SuperNova, Dual, Mo at zero, Atlas
Data collection method	ω scan
Absorption correction	multi-scan
No. of measured, independent and observed reflections	22540, 2568, 2413
Criterion for observed reflections	$F^2 > 2.0\ \sigma(F^2)$
R_{int}	0.0384
θ range (°)	3.704–74.806
h range	−18–19
k range	−17–18
l range	−35–31
**Refinement**	
Refinement method	full-matrix least-squares refinement on F^2
R (on F_{obs}), wR (on F_{obs}), S	0.0438, 0.1148, 1.001
No. of contributing reflections	2568
No. of parameters	155
No. of restraints	1
$(\Delta/\sigma)_{max}$, $(\Delta/\sigma)_{ave}$	< 0.001, < 0.001
$\Delta\rho_{max}$, $\Delta\rho_{min}$ (eÅ^{-3})	0.301, −0.488

3 NMR Measurements

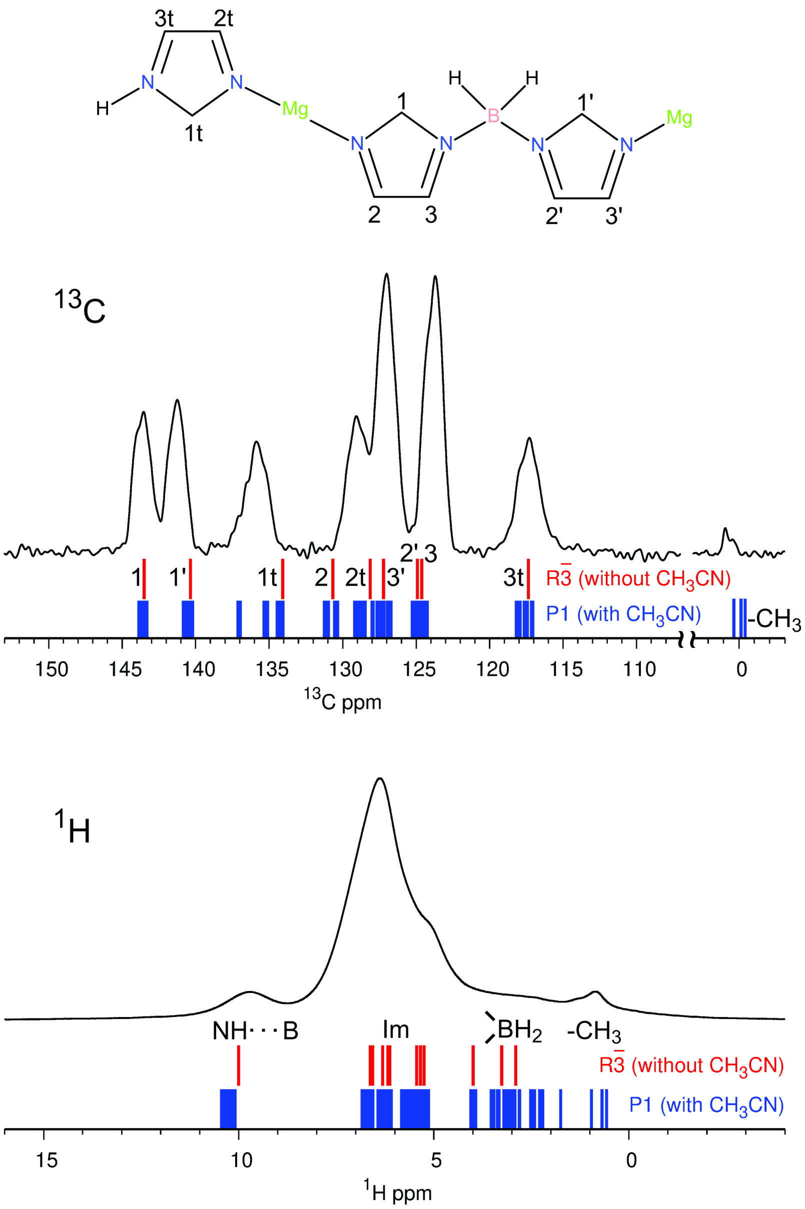

Figure S4: ^{1}H-^{13}C CPMAS and ^{1}H MAS NMR spectra of the acetonitrile solvate (black solid lines) and calculated ^{13}C and ^{1}H isotropic chemical shifts (short vertical red and blue lines). Chemical shifts were calculated using two different structural models, one with high-symmetry and without acetonitrile molecules (values marked with red bars) and one with low symmetry and with acetonitrile molecules (values marked with blue bars).

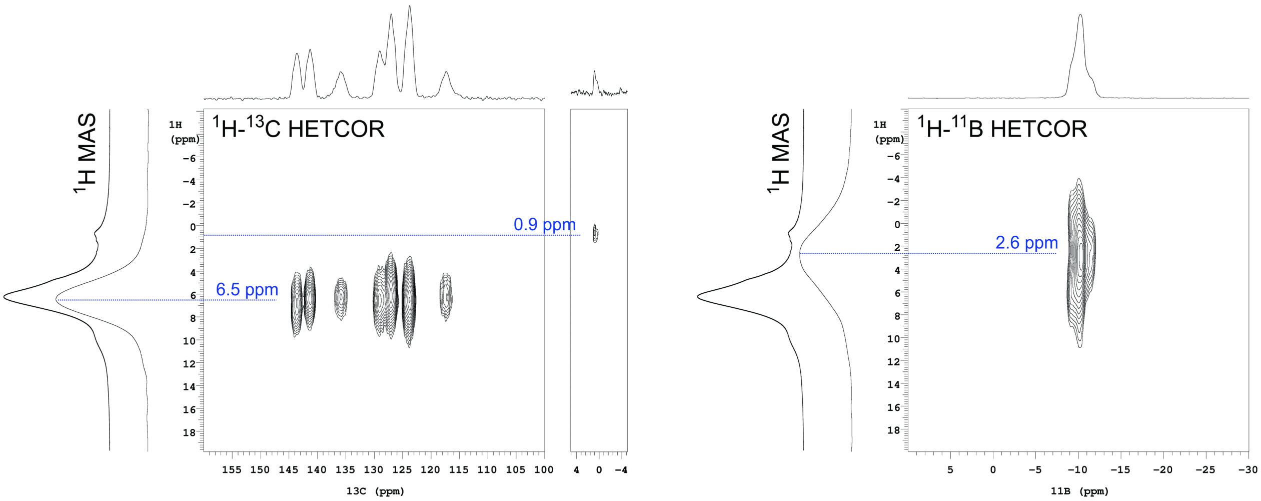

Figure S5: Two-dimensional ^{1}H-^{13}C and ^{1}H-^{11}B HETCOR NMR spectra of the acetonitrile solvate. The spectra show, which hydrogen atoms are close to which carbon or boron atoms. In both spectra the projection onto the ^{1}H axis is compared with the ^{1}H MAS NMR spectrum.

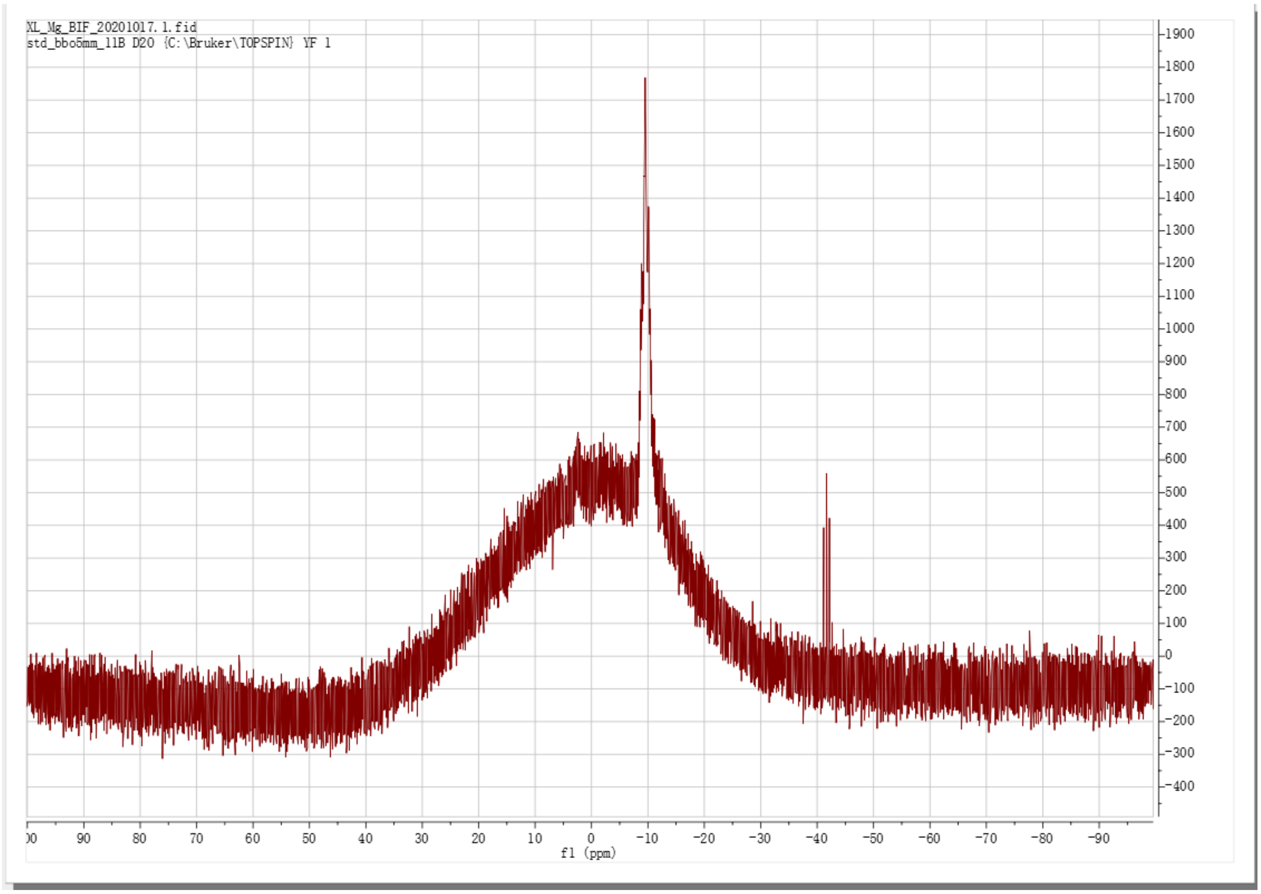

Figure S6: ^{11}B NMR spectrum of the imidazole solvate, obtained from imidazole melt, in D_2O. It reveals only a peak at -9.58 ppm for BH_2 group (bisubstituted borohydride) and a small amount of the BH_4 impurity at -41.69 ppm.

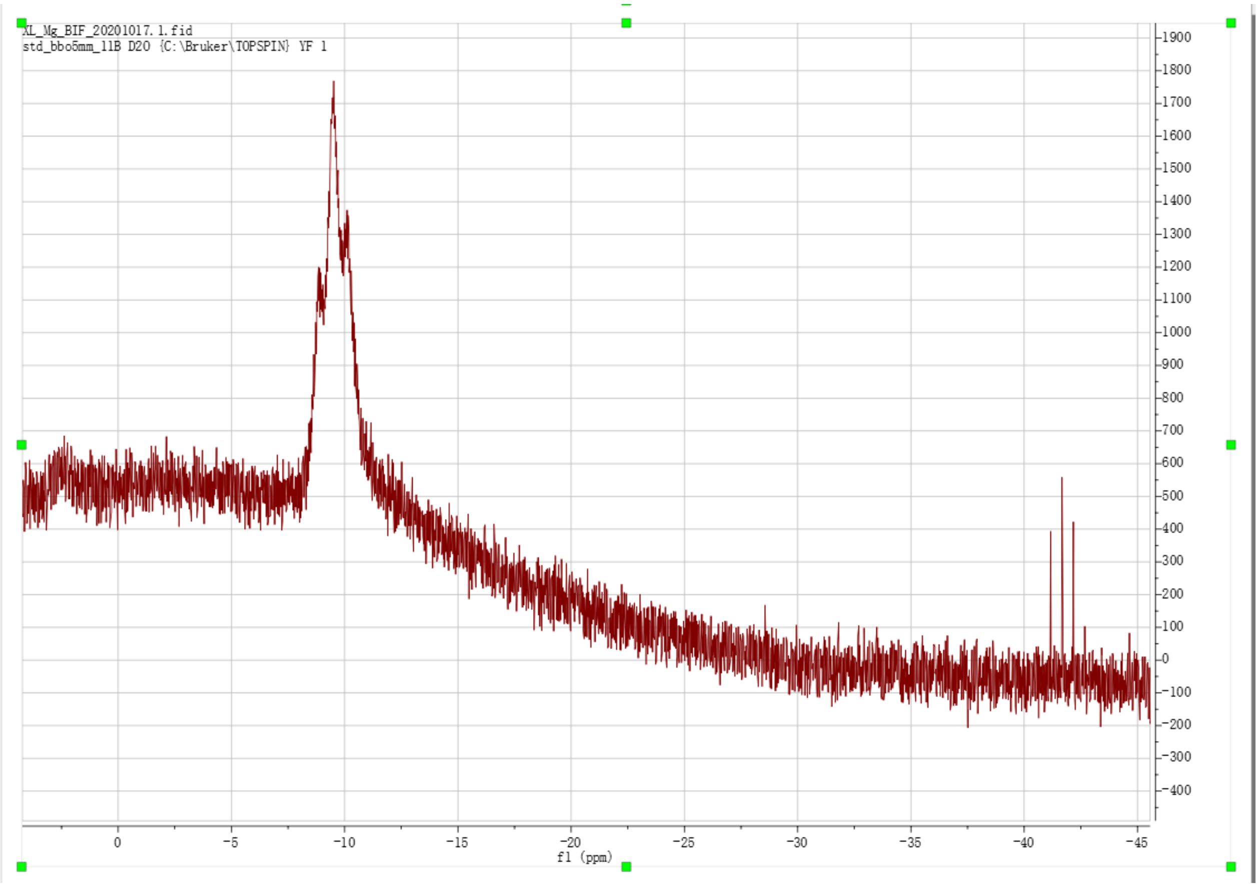

Figure S7: ^{11}B NMR spectrum of the imidazole solvate showing a smaller region in detail.

4 Sorption Analysis

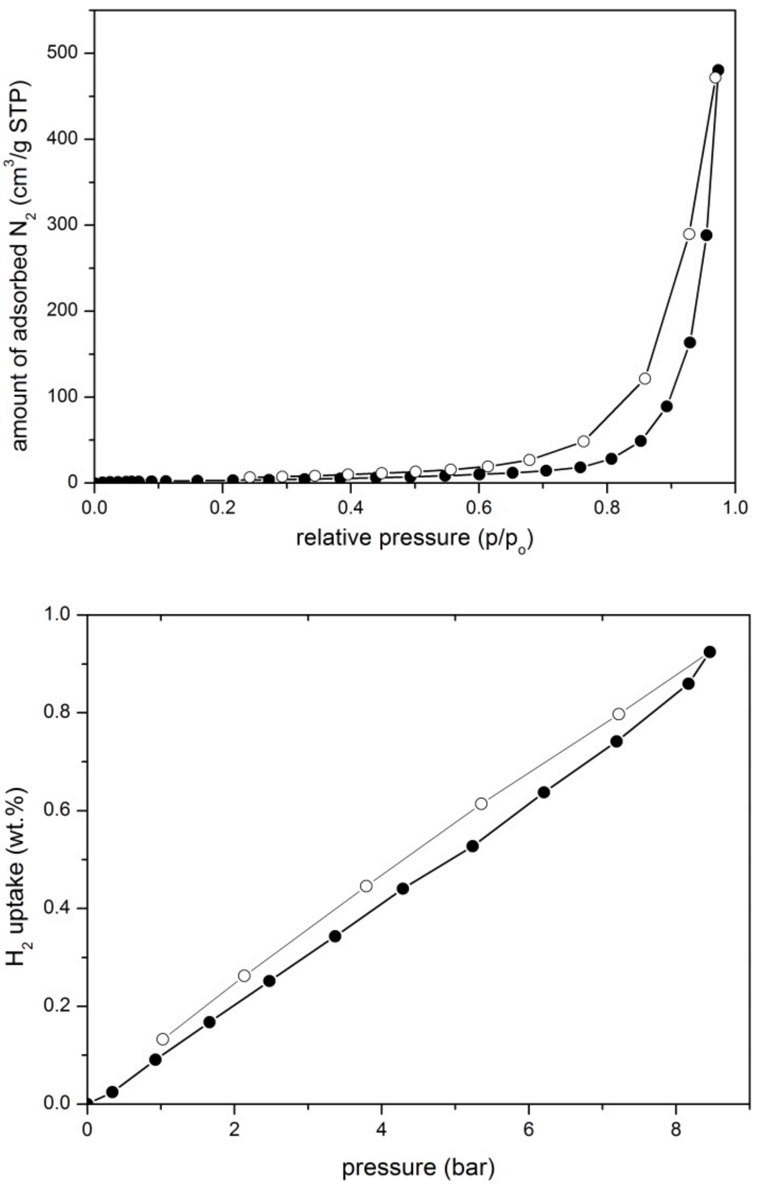

Figure S8: N_2 (top) and H_2 (bottom) isotherms of $[Mg_3\{(Im)BH_2(Im)\}_6(ImH)_6] \cdot CH_3CN$ material measured at 77 K.

5 DFT Calculations

Table S4: Comparison of unit cell volume V and unit cell vector lengths a and c, obtained with periodic DFT calculations using different functionals. ΔV, Δa and Δc represent discrepancies of calculated parameters relative to experimental values. DC stands for dispersion correction. Experimental data was obtained from a single crystal at temperature 150 K. Compared functionals are revised Perdew-Burke-Ernzerhof (RP), Perdew-Burke-Ernzerhof (PE) and Perdew-Burke-Ernzerhof revised for solids (PS). Initial configuration for DFT calculations was experimental structure with a single acetonitrile molecule placed at each place of disorder.

**Parameter**	**RP**	**PE**	**PS**	**RP (no DC)**	**Experimental**
V [Å^3]	5679	5605	5342	6273	5661.27
a [Å]	15.19	15.09	15.02	15.81	15.1942
c [Å]	28.40	28.37	28.14	28.96	28.3157
ΔV	0.3 %	−1.0 %	−5.7 %	11 %	
Δa	0.02 %	−0.67 %	−1.2 %	4.0 %	
Δc	0.29 %	0.20 %	−0.62 %	2.3 %	

6 Synchrotron Powder Diffraction

6.1 $[Mg_3\{(Im)BH_2(Im)\}_6(ImH)_6]\cdot ImH$

The material synthesized in the imidazole melt was expected to be a solvent-free form of the title compound. This hypothesis was tested by Rietveld refinement. A structural model was constructed containing only the trinuclear complex $[Mg_3\{(Im)BH_2(Im)\}_6(ImH)_6]$ as determined by single-crystal diffraction of the acetonitrile solvate, leaving voids in place of the solvate molecules. The complex was included in the structure as a Z-matrix. Its bond lengths, angles and dihedral angles were refined within the limits observed in similar known structural fragments. Surprisingly, the obtained fit was quite poor. In particular, the peaks at low angles exhibited large intensity mismatch (Figure S9). It was evident from the difference Fourier map that this was due to the lack of electron density between the ends of the trinuclear moieties – in the voids occupied by disordered acetonitrile molecules in the acetonitrile solvate form.

Since the solvent molecules in the crystal structure are located near the point of high symmetry ($\bar{3}$), which causes the electron density to appear shapeless due to symmetry-induced disorder, it was not possible to discern a molecular shape from the electron density map. The only logical possibility to explain the missing electron density was the presence of imidazole molecules in the role of the solvent. Therefore, the imidazole molecule was constructed in the form of a Z-matrix and added to the structural model. Refined parameters included its orientation, position, atomic displacement parameter, equal for all atoms of the molecule, and the population parameter. Molecular geometry of imidazole was not refined. Bond lengths, angles and dihedral angles were determined based on known structures.

Refinement details are given in Table S5 and the Rietveld plot is presented in Figure S10.

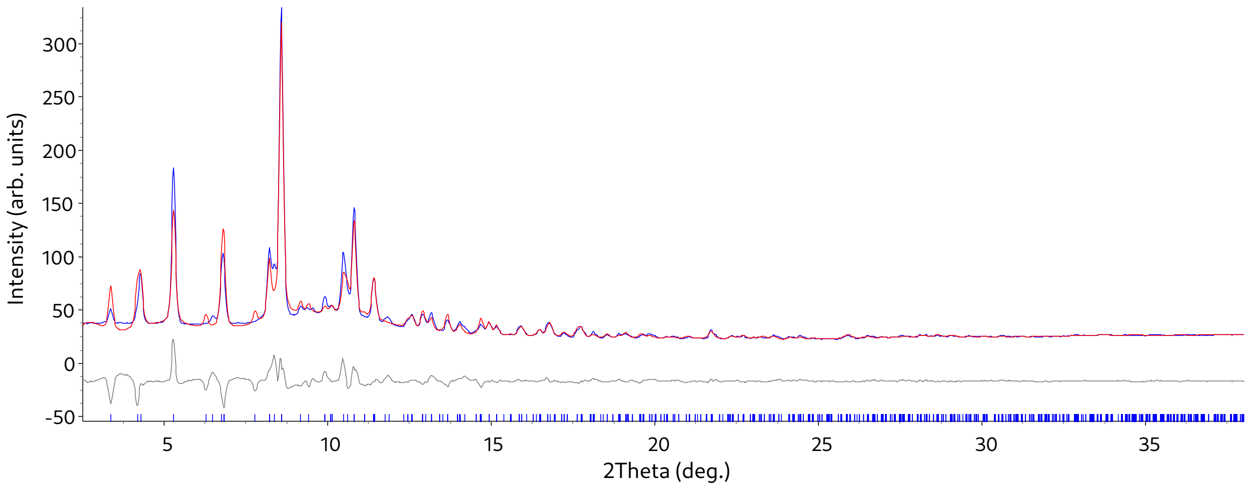

Figure S9: Rietveld fit of the powder pattern of the imidazole solvate without accounting for the presence of the solvent.

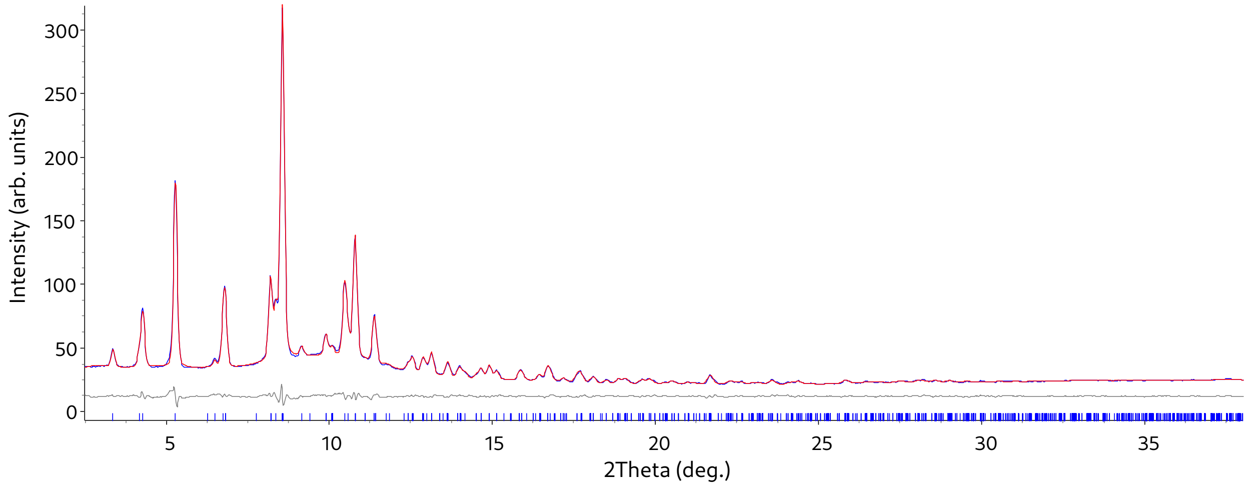

Figure S10: Rietveld fit of a synchrotron powder pattern containing only the imidazole solvate at 80 °C.

Table S5: Crystal data, data collection and refinement details of $[Mg_3\{(Im)BH_2(Im)\}_6(ImH)_6] \cdot ImH$.

**Crystal data**	
Formula	$C_{57}H_{76}B_6Mg_3N_{38}$
M_r	1504.05
Cell setting, space group	Trigonal, $R\bar{3}$
a	15.3943(5) Å
c	28.4441(18) Å
V	5837.7(5) Å^3
Z	3
D_x (Mg m^{-3})	1.284
Sample form, color	powder, white
**Data collection**	
T	353(1) K
Radiation type, wavelength	synchrotron, 0.7093 Å
Facility, beamline	ESRF, SNBL
Measured/used range	1.4–51.0/2.5–38.0 °2θ
Used range, d-spacing	16.26–1.089 Å
No. of reflections	1061
**Refinement**	
Refinement method	Rietveld
R_{exp}, R_{wp}	0.50 %, 1.58 %
R'_{exp}, R'_{wp}	2.44 %, 7.76 %
R_{Bragg}	0.5 %
No. of profile parameters	29
No. of structural parameters (Z-matrix)	61

6.2 $Mg(BHIm_3)_2$

The successful structure solution of $Mg(BHIm_3)_2$ was based on the following considerations and trials.

1. It is likely that the solvent molecules leave the material during structural rearrangement at elevated temperature. The other possibility would be that they react with the $[Mg_3\{(Im)BH_2(Im)\}_6(ImH)_6]$ moieties, but the former was considered more likely as a gradual trend of decreasing solvent population parameter with increasing temperature was observed.

2. The building moiety $[Mg_3\{(Im)BH_2(Im)\}_6(ImH)_6]$ is too large to fit into the unit cell of the new structure, so it must be disintegrated. The molar mass of the moiety is about 1362 g/mol, and even if only one such moiety were present in the unit cell with volume of about 1170 $\mathring{A}^3$, the density would be much too high (more than 1.9 g/cm^3). The expected density of the new phase, composed of the same material as the trinuclear building moiety, is about 1.3 g/cm^3.

3. The main consideration was focused on identifying the weakest points in the molecular structure of $[Mg_3\{(Im)BH_2(Im)\}_6(ImH)_6]$ that would react or disintegrate first upon heating. A possible answer was found following the idea we had when planning the synthesis of the material – as many potentially reactive negatively charged hydrides as possible should remain bound to boron, thus having the opportunity to react with positively charged hydrogens and evolve hydrogen gas by comproportionation. In the $[Mg_3\{(Im)BH_2(Im)\}_6(ImH)_6]$ unit, there are 12 negatively charged hydrides, 54 hydrogens bonded to carbon atoms, and 6 hydrogens bonded to nitrogen atoms of the terminal imidazoles (Figure 1 in the main text). From these facts, it was quite straightforward to conclude that 6 hydrides bonded to boron atoms and 6 hydrogens bonded to nitrogen atoms could react to from 6 hydrogen molecules. At the same time, the B−H and N−H bonds would be compensated by the formation of a new B−N bond. In this way, a new building unit, namely $BHIm_3$ with a single negative charge, would be formed. Thus, the simple formula of the product, maintaining charge balance, would be $Mg(BHIm_3)_2$ and from one moiety of $[Mg_3\{(Im)BH_2(Im)\}_6(ImH)_6]$ three such moieties would be formed after the evolution of 6 hydrogen molecules.

4. There are several known structures containing the $BHIm_3$ moiety (for example MOQJAP and MOXKEQ in the CSD), so it was possible to construct a fairly realistic Z-matrix for such a structural fragment. It was also quite realistic to expect that the magnesium ions would be coordinated octahedrally by six "free" (not bound to boron) imidazolate nitrogen atoms.

5. From the molar mass of $Mg(BHIm_3)_2$ (about 450 g/mol), the volume of the unit cell (about 1170 $\mathring{A}^3$), and the expected density (about 1.3 g/cm^3), it was concluded that there must be two formula units in the unit cell (two magnesium ions and four $BHIm_3$ moieties).

6. The shape of the unit cell is consistent with either hexagonal or trigonal crystallographic system. However, based on the above, the trigonal system was chosen as far more probable. Namely, the $BHIm_3$ moiety is pyramidal and was expected to be positioned on the threefold axis (B and H atoms on the axis running through the point ($\frac{1}{3}$, $\frac{2}{3}$, z), and one of the three imidazolates being part of the asymmetric unit, while the other two are its symmetry equivalents). Magnesium was expected to be lie on the other threefold axis passing through the origin. The positions on the axis of both Mg and $BHIm_3$, rotation of the latter around the threefold axis, as well as some internal coordinates (bond angle H−B−N and dihedral angle describing the rotation of the imidazolate about the B−N bond) were searched to achieve reasonable packing and coordination of magnesium.

7. Several trigonal space groups were possible, and initially some trials to determine approximate positions of the structural fragments were performed with the lowest possible symmetry (P3), in the hope that constraining the fragments (Mg and $BHIm_3$) to the threefold axes would be sufficient to keep the structure reasonable, while allowing the fragments some degree of freedom: movement along the threefold axes, $BHIm_3$ rotation around the axis, flexible H−N−B angle and imidazolate rotation. It was expected that refinement against the measured powder pattern will move the fragments to correct experimental positions and conformations. The true symmetry of the structure would then be determined from the lower-symmetry structure solution.

8. Described low-symmetry approach was not successful and the structures obtained were not reproducible. Changing the symmetry to P$\bar{3}$ did not yield expected results and therefore it was necessary to find the highest possible symmetry compatible with the fragments and the overall stoichiometry. Analysis of the extinction rules showed that they are consistent with a c glide plane, perpendicular to the longer diagonal of the ab plane. Thus, the most symmetric space group possible was P$\bar{3}$1c. In this space group, the asymmetric unit consists only of Mg at the origin (coordinates [0, 0, 0], Wyckoff position 2b), boron and hydrogen on the other threefold axis (coordinates [$\frac{1}{3}$, $\frac{2}{3}$, z], Wyckoff position 4f), and atoms of an imidazolate ring on general positions (Wyckoff 12i). The symmetry generates the other magnesium, three more B and H atoms and, eleven more imidazolates. The z coordinate of $BHIm_3$, its rotation about the axis and the internal coordinates, as described above, were refined.

9. When this space group was applied, the structure readily converged to a reasonable arrangement (Figure S11) with acceptably good fit of the measured powder pattern (Figure S12). Refinement details are given in Table S6.

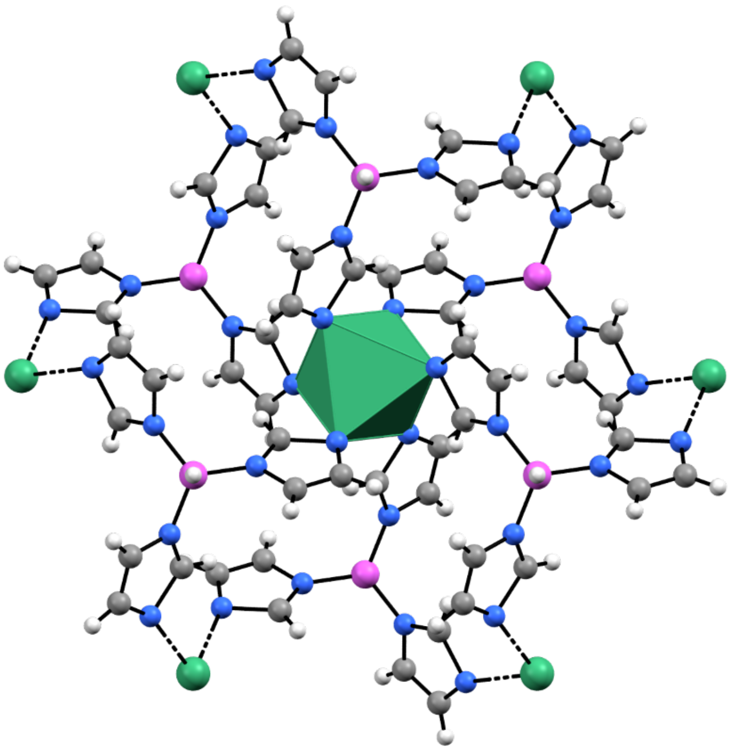

Figure S11: Octahedral coordination of Mg atoms in the structure of $Mg(BHIm_3)_2$.

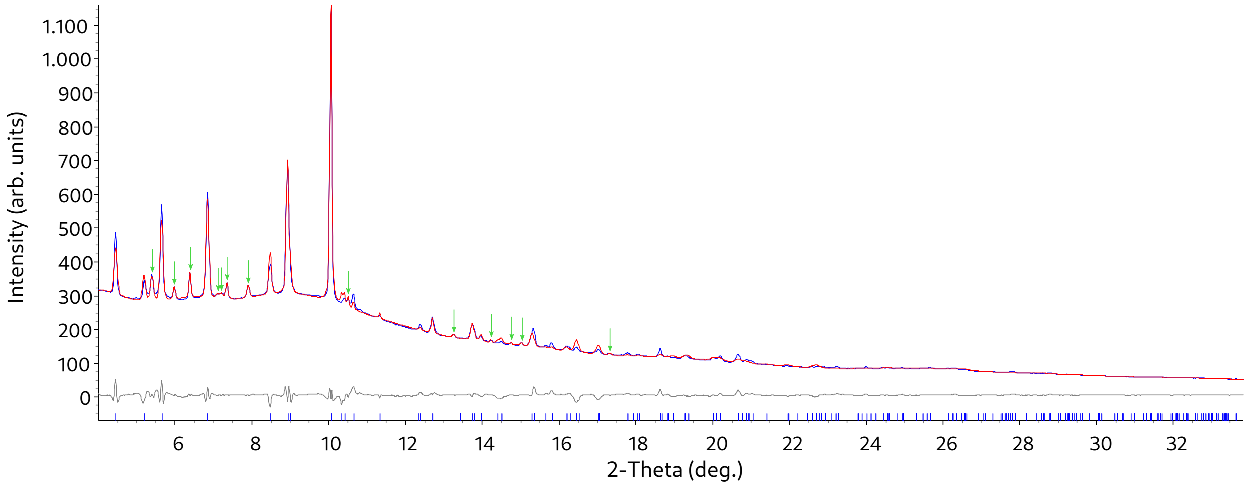

Figure S12: Rietveld fit of $Mg(BHIm_3)_2$. Individually fitted peaks of an unknown impurity are marked with green arrows.

Table S6: Crystal data, data collection and refinement details of $Mg(BHIm_3)_2$.

**Crystal data**	
Formula	$C_{18}H_{20}B_2MgN_{12}$
M_r	450.39
Cell setting, space group	Trigonal, P$\bar{3}$1c
a	8.7338(9) Å
c	17.621(2) Å
V	1164.1(3) Å^3
Z	2
D_x (Mg m^{-3})	1.285(1)
Sample form, color	powder, white
**Data collection**	
T	298(1) K
Radiation type, wavelength	synchrotron, 0.686 83 Å
Facility, beamline	ESRF, SNBL
Measured/used range	0.01–42.15/4.00–33.81 °2θ
Used range, d-spacing	9.83–1.18 Å
No. of reflections	258
**Refinement**	
Refinement method	Rietveld
R_{exp}, R_{wp}	0.32 %, 2.53 %
R'_{exp}, R'_{wp}	2.61 %, 20.29 %
R_{Bragg}	1.45 %
No. of profile parameters	33
No. of structural parameters (Z-matrix)	24
No. of impurity peaks fitted individually	13
